# The origin of Eastern European Jews revealed by autosomal, sex chromosomal and mtDNA polymorphisms

**DOI:** 10.1186/1745-6150-5-57

**Published:** 2010-10-06

**Authors:** Avshalom Zoossmann-Diskin

**Affiliations:** 1 Department of Haematology and Genetic Pathology, School of Medicine, Flinders University, Adelaide, Australia; 2 Department of Human Genetics, Sackler Faculty of Medicine, Tel-Aviv University, Israel; 3 Current Address: Blood Bank, Sheba Medical Center, Ramat-Gan 52621, Israel

## Abstract

**Background:**

This study aims to establish the likely origin of EEJ (Eastern European Jews) by genetic distance analysis of autosomal markers and haplogroups on the X and Y chromosomes and mtDNA.

**Results:**

According to the autosomal polymorphisms the investigated Jewish populations do not share a common origin, and EEJ are closer to Italians in particular and to Europeans in general than to the other Jewish populations. The similarity of EEJ to Italians and Europeans is also supported by the X chromosomal haplogroups. In contrast according to the Y-chromosomal haplogroups EEJ are closest to the non-Jewish populations of the Eastern Mediterranean. MtDNA shows a mixed pattern, but overall EEJ are more distant from most populations and hold a marginal rather than a central position. The autosomal genetic distance matrix has a very high correlation (0.789) with geography, whereas the X-chromosomal, Y-chromosomal and mtDNA matrices have a lower correlation (0.540, 0.395 and 0.641 respectively).

**Conclusions:**

The close genetic resemblance to Italians accords with the historical presumption that Ashkenazi Jews started their migrations across Europe in Italy and with historical evidence that conversion to Judaism was common in ancient Rome. The reasons for the discrepancy between the biparental markers and the uniparental markers are discussed.

**Reviewers:**

This article was reviewed by Damian Labuda (nominated by Jerzy Jurka), Kateryna Makova and Qasim Ayub (nominated by Dan Graur).

## Background

The genetic affinities of the Jewish populations have been studied since the early days of genetics, yet the origin of these populations is still obscure. Some of the studies, trying to establish the origins of the Jewish populations with autosomal markers, claimed that the Jewish populations have a common origin, but others concluded that the Jews are a very diverse group. This corpus of studies has already been critically reviewed [1].

The origin of Eastern European Jews, (EEJ) by far the largest and most important Ashkenazi population, and their affinities to other Jewish and European populations are still not resolved. Studies that compared them by genetic distance analysis of autosomal markers to European Mediterranean populations revealed that they are closer to Europeans than to other Jewish populations [1-3].

EEJ are the largest and most investigated Jewish community, yet their history as Franco-German Jewry is known to us only since their appearance in the 9th century, and their subsequent migration a few hundred years later to Eastern Europe [4,5]. Where did these Jews come from? It seems that they came to Germany and France from Italy [5-8]. It is also possible that some Jews migrated northward from the Italian colonies on the northern shore of the Black Sea [9]. All these Jews are likely the descendents of proselytes. Conversion to Judaism was common in Rome in the first centuries BC and AD. Judaism gained many followers among all ranks of Roman Society [10-13].

The aim of this study is to establish the likely origin of this major Jewish population by using a larger dataset of autosomal markers, and compare the results to analyses based on the available data for the X and Y chromosomes and for mtDNA.

## Methods

Six Jewish populations: EEJ, Moroccan Jews, Iraqi Jews. Iranian Jews, Yemenite Jews and Ethiopian Jews, which have been studied for all the autosomal markers used in this study, are included in the analysis. EEJ are defined on the basis of history as those Jews originating from the areas of the Polish-Lithuanian Kingdom and their descendants in bordering regions, encompassing the territories of Russia, Poland, the Baltic States, Belarus, Moldavia, Moldova (the north-eastern part of Romania) and the Ukraine. The Data on the non-autosomal markers were also available for other Jewish populations: Bulgarian Jews (X, mtDNA), Turkish Jews (X, mtDNA), Tunisian Jews (mtDNA), Libyan Jews (Y, mtDNA) and Djerban Jews (Y).

The seventeen autosomal markers are: AK, ADA, PGM1, PGD, ACP, ESD, GPT, HP, GC, J311 MspI & MetH TaqI (both on chromosome 7 near the CF locus), FV G1691A, FII G20210A, MTHFR C677T, CBS 844ins68, ACE ID and PAH XmnI. All the markers are unique-event-polymorphisms, and apart from two insertions (CBS 844ins68, ACE ID) are all SNPs. The first nine markers are polymorphisms of red cell enzymes and serum proteins, and were typed mostly by protein electrophoresis, but the variation at the protein level is directly related in a 1:1 manner to the SNP variation at the DNA level. Indeed, some of the results for the Jewish populations were obtained by PCR methods [1,14]. The polymorphism of the remaining eight markers can only be detected at the DNA level. J311 MspI and MetH TaqI were typed in all the populations including the Israeli populations (unpublished results) by Southern blotting and hybridization [15,16]. The other 6 markers were typed in the Israeli populations by PCR methods. The data on FV G1691A, FII G20210A, MTHFR C677T and CBS 844ins68 have already been published [3,17]. The data on ACE ID and PAH XmnI are still unpublished. These polymorphisms were typed according to the methods of Rigat et al. [18] and Goltsov et al. [19] respectively. Allele frequencies for all the populations are given in Additional file 1: tables S1-4. Table S2 (Additional file 1) presents four markers on both sides of the CF locus. Because of the linkage between them, I chose to use only the two most distal markers, which are separated by a few centimorgans. Haplogroup frequencies of the non-recombining Y chromosome (NRY), the X chromosome (dystrophin locus, dys44, on Xp21.3) and mtDNA are given in Additional file 1: tables S5, S6 and S7 respectively.

Gower (cited in [20]) recommends, that for microevolutionary studies, when sample sizes are quite variable and gene frequencies do not differ greatly, Sanghvi's G2 [21] would be the most appropriate, and this is the measure I used. Distances were also calculated with Nei's [22] formula and the results were very similar (r = 0.990, genetic distance matrix not shown). The neighbor joining tree was computed by PHYLIP 3.66. Since it does not calculate Sanghvi's G2, I used Reynolds et al. distance [23], which is also based on the assumption that gene frequencies change by genetic drift alone, solely for the calculation of the tree (genetic distance matrix not shown). The significance of nodes in the tree and the standard errors of the genetic distances were computed by bootstrapping 10,000 times. Multidimensional scaling plots and Mantel tests for correlation between genetic distance matrices and between them and matrices of geographic distances were computed by NTSYS 1.70. Geographic distances were calculated as great circle distances between the capitals of the countries of origin of the populations (Warsaw was chosen for EEJ). Mantel test significance was assessed by 10,000 permutations.

## Results

**Figure 1:**
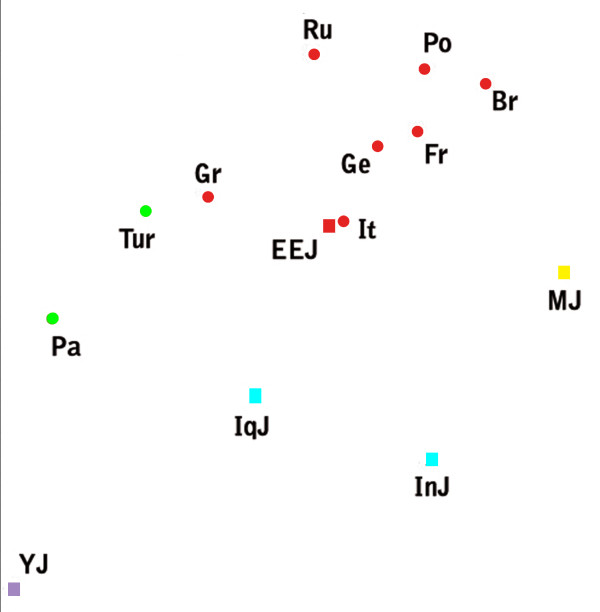
**A multidimensional scaling plot of the autosomal genetic distance matrix excluding Ethiopian Jews**. Stress = 0.100. Populations names are: EEJ - Eastern European Jews, IqJ - Iraqi Jews, InJ - Iranian Jews, MJ - Moroccan Jews, YJ - Yemenite Jews, Pa - Palestinians, Tur - Turks, Gr - Greeks, It - Italians, Ge - Germans, Br - British, Fr - French, Ru - Russians, Po - Poles. Squares represent Jews and circles non-Jews. Colour indicates geographic region: red - Europe, green - Eastern Mediterranean, blue - Iran-Iraq, purpule - Arabian peninsula, yellow - North-Africa.

**Figure 2:**
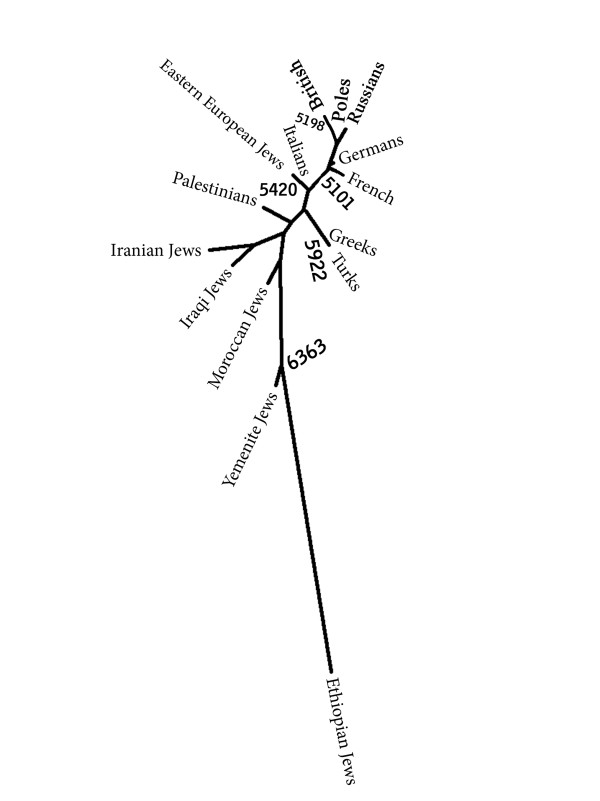
**A neighbor joining tree based on the autosomal polymorphisms**. A number next to a node indicates the majority bootstrap support for that node out of 10,000 repetitions.

The autosomal genetic distances (table 1) do not show any particular resemblance between the Jewish populations. EEJ are closer to Italians in particular and to Europeans in general than to the other Jewish populations. All of the distances, apart from one, differ from zero by more than twice their standard error. A difference between two distances can be considered meaningful, if it is more than twice their largest standard error. The differences between the distance of EEJ from Italians and their distances from the other Jewish populations are meaningful according to this criterion, and the same is also true for all the Non-Jewish populations except for Greeks and Russians. In fact the distance between EEJ and Italians is the smallest distance in the matrix. A multidimensional scaling plot of the genetic distance matrix (figure 1) captures the proximity of EEJ to Italians and other European populations. The same is also true for the neighbor joining tree (figure 2). It should be noted that multidimensional scaling plots are a way to present graphically the intricate relationships of genetic distance matrices. As such they are necessarily less accurate than the matrices on which they are based. In order to understand the genetic affinities of a particular population, one must examine its distances in the matrix itself, not in the plot. The same also applies to the neighbor joining tree. The bootstrap values indicate the robustness of the clustering, but not the significance of individual genetic distances.

**Table 1:** Autosomal genetic distance matrix (×1000) (standard errors above the diagonal)

	1	2	3	4	5	6	7	8	9	10	11	12	13	14	15
**1) EEJ**		103	94	52	180	348	76	57	38	11	35	73	42	94	58

**2) Iraqi Jews**	277		68	131	87	330	58	147	117	87	64	125	138	141	99

**3) Iranian Jews**	275	218		131	118	391	125	112	97	105	119	149	142	146	139

**4) Moroccan Jews**	243	330	325		148	263	105	115	89	36	66	71	55	80	78

**5) Yemenite Jews**	498	366	335	447		263	87	104	92	162	133	123	114	155	168

**6) Ethiopian Jews**	1240	1127	1004	809	696		233	322	333	349	396	373	341	381	463

**7) Palestinians**	277	223	425	298	323	972		43	44	60	65	131	63	87	122

**8) Turks**	170	243	305	314	400	1244	182		15	54	56	113	117	64	68

**9) Greeks**	105	270	316	311	356	1246	202	56		36	38	83	76	42	52

**10) Italians**	44	243	255	167	452	1083	231	157	101		25	48	34	81	40

**11) Germans**	131	268	294	237	511	1067	299	179	148	71		25	19	34	12

**12) British**	238	395	373	239	592	977	434	332	267	151	53		41	46	13

**13) French**	144	339	398	216	545	974	288	265	192	91	48	75		59	33

**14) Russians**	230	420	430	289	513	1144	375	175	139	193	102	112	134		25

**15) Poles**	195	405	365	264	600	1204	465	255	197	139	50	46	102	66	

**Figure 3:**
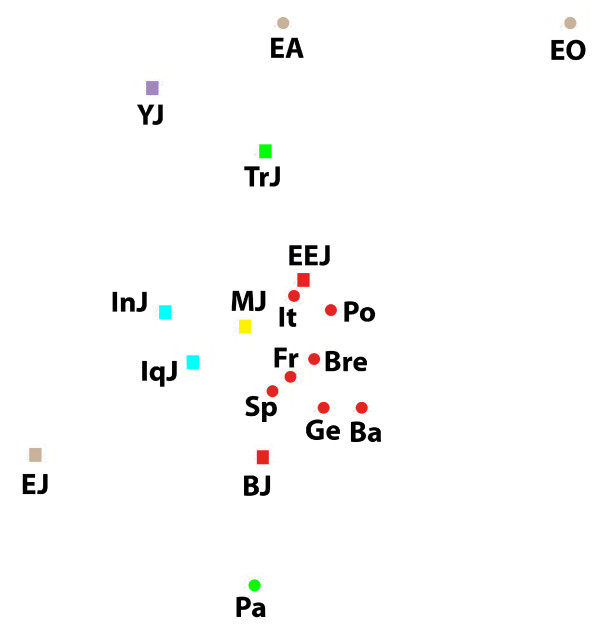
**A multidimensional scaling plot of the X-chromosomal genetic distance matrix**. Stress = 0.125. Populations names are: EEJ - Eastern European Jews, IqJ - Iraqi Jews, InJ - Iranian Jews, MJ - Moroccan Jews, YJ - Yemenite Jews, EJ - Ethiopian Jews, BJ - Bulgarian Jews, TrJ - Turkish Jews, Pa - Palestinians, It - Italians, Ge - Germans, Po - Poles, Fr - French, Bre - Bretons, Sp - Spaniards, Ba - Basques, EO - Ethiopians Oromo, EA - Ethiopians Amhara. Squares represent Jews and circles non-Jews. Colour indicates geographic region: red - Europe, green - Eastern Mediterranean, blue - Iran-Iraq, purpule - Arabian peninsula, yellow - North-Africa, brown - Ethiopia.

**Figure 4:**
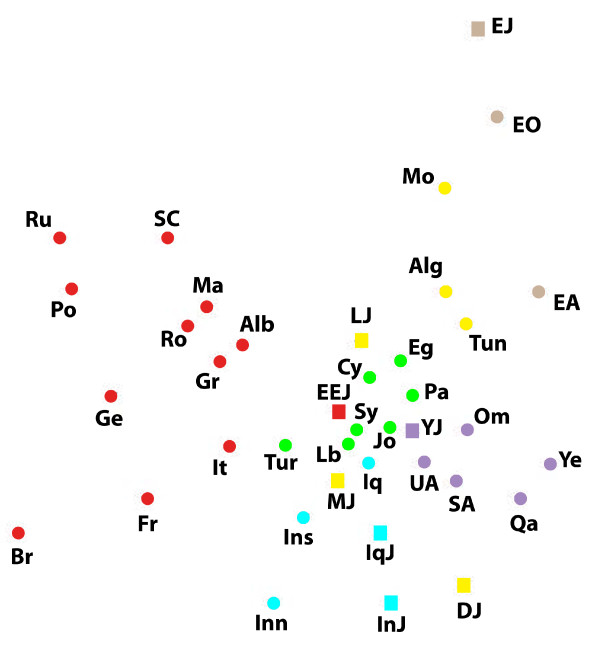
**A multidimensional scaling plot of the Y-chromosomal genetic distance matrix**. Stress = 0.133. Populations names are: EEJ - Eastern European Jews, IqJ - Iraqi Jews, InJ - Iranian Jews, MJ - Moroccan Jews, LJ - Libyan Jews, DJ - Djerban Jews, YJ - Yemenite Jews, EJ - Ethiopian Jews, Pa - Palestinians, It - Italians, Fr - French, Br - British, Ge - Germans, Ru - Russians, Po - Poles, SC - Serbo-Croats, Alb - Albanians, Gr - Greeks, Ma - Macedonians, Ro - Romanians, Tur - Turks, Inn - Iranians-North, Ins - Iranians-South, Iq - Iraqis, Cy - Cypriots, Sy - Syrians, Lb - Lebanese, Jo - Jordanians, SA - Saudi-Arabians, Qa - Qataris, UA - United Arab Emirates, Om - Omanis, Ye - Yemenites, Eg - Egyptians, Mo - Moroccans, Alg - Algerians, Tun - Tunisians, EO - Ethiopians Oromo, EA - Ethiopians Amhara. Squares represent Jews and circles non-Jews. Colour indicates geographic region: red - Europe, green - Eastern Mediterranean, blue - Iran-Iraq, purpule - Arabian peninsula, yellow - North-Africa, brown - Ethiopia.

**Figure 5:**
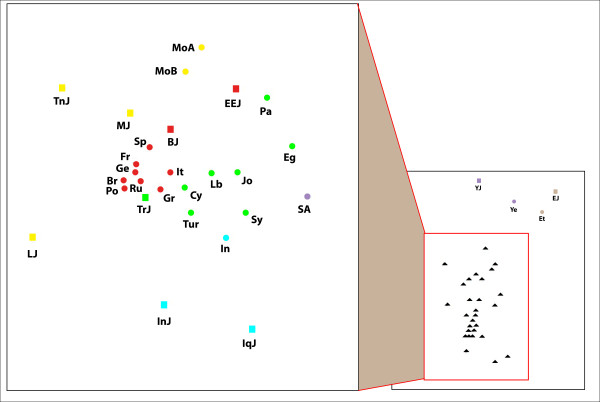
**A multidimensional scaling plot of the mtDNA genetic distance matrix**. Stress = 0.110 for the outer plot and 0.161 for the inner one. Populations names are: EEJ - Eastern European Jews, IqJ - Iraqi Jews, InJ - Iranian Jews, MJ - Moroccan Jews, LJ - Libyan Jews, TnJ - Tunisian Jews, BJ - Bulgarian Jews, TrJ - Turkish Jews, YJ - Yemenite Jews, EJ - Ethiopian Jews, Pa - Palestinians, It - Italians, Fr - French, Br - British, Ge - Germans, Ru - Russians, Po - Poles, Sp - Spaniards, Gr - Greeks, Tur - Turks, In - Iranians, Cy - Cypriots, Sy - Syrians, Lb - Lebanese, Jo - Jordanians, SA - Saudi-Arabians, Ye - Yemenites, Eg - Egyptians, MoA - Moroccan Arabs, MoB - Moroccan Berbers, Et - Ethiopians. Squares represent Jews and circles non-Jews. Colour indicates geographic region: red - Europe, green - Eastern Mediterranean, blue - Iran-Iraq, purpule - Arabian peninsula, yellow - North-Africa, brown - Ethiopia.

X-chromosomal haplogroups demonstrate the same relatedness of EEJ to Italians and other Europeans (table 2, figure 3). In contrast, according to the Y-chromosomal haplogroups EEJ are closest to the non-Jewish populations of the Eastern Mediterranean (table 3, figure 4). MtDNA shows a mixed pattern where EEJ are about equally close to Moroccan Jews, Palestinians, Italians and Bulgarian Jews, but overall are more distant from most populations and hold a marginal position in the MDS plot, rather than a central one like in the other plots (table 4, figure 5).

**Table 2:** X chromosomal genetic distance matrix (×1000)

1) EE Jews	1	2	3	4	5	6	7	8	9	10	11	12	13	14	15	16	17
**2) Iraqi Jews**	402																

**3) Iranian Jews**	497	351															

**4) Moroccan Jews**	302	211	480														

**5) Yemenite Jews**	555	406	512	439													

**6) Ethiopian Jews**	533	617	683	676	709												

**7) Bulgarian Jews**	409	276	440	299	611	672											

**8) Turkish Jews**	288	519	474	452	403	599	625										

**9) Palestinians**	573	506	512	464	666	754	350	712									

**10) Italians**	223	374	488	184	493	741	337	395	478								

**11) Germans**	263	483	497	358	715	701	318	518	502	282							

**12) Poles**	233	482	531	336	570	741	406	476	484	235	266						

**13) Basques**	311	597	548	513	827	702	378	479	503	369	349	359					

**14) Spaniards**	252	385	457	313	609	554	297	406	487	334	315	365	337				

**15) French**	313	332	454	284	649	706	206	401	483	285	308	347	249	244			

**16) Bretons**	186	410	483	386	615	611	288	376	492	288	238	246	234	219	162		

**17) Ethiopians Oromo**	771	918	892	906	977	1243	847	745	1002	753	816	797	840	840	717	727	

**18) Ethiopians Amhara**	490	618	619	504	471	798	695	433	702	449	614	490	680	579	555	524	791

**Table 3:** Y chromosomal genetic distance matrix (×1000)*

1) EEJ	1	2	3	4	5	6	7	8	9	10	11	12	13	14	15	16	17	18	19	20	21	22	23	24	25	26	27	28	29	30	31	32	33	34	35	36	37	38
**2) IqJ**	341																																					

**3) InJ**	574	236																																				

**4) MJ**	245	335	764																																			

**5) LJ**	242	626	863	465																																		

**6) DJ**	582	813	1025	667	402																																	

**7) YJ**	185	244	472	304	418	545																																

**8) EJ**	1296	1373	1444	1386	1308	1685	1278																															

**9) Pa**	192	469	728	362	351	411	215	1254																														

**10) It**	357	720	1022	332	538	928	669	1427	611																													

**11) Ge**	815	1209	1356	933	1194	1614	1179	1644	1196	424																												

**12) Br**	1233	1504	1801	1060	1494	1727	1475	1860	1474	499	398																											

**13) Fr**	754	1053	1177	749	1034	1299	971	1622	1043	307	399	346																										

**14) Ru**	1150	1303	1299	1384	1504	1811	1498	1737	1406	1159	595	1364	1255																									

**15) Po**	1030	1388	1430	1316	1359	1740	1388	1687	1337	971	388	1119	1058	185																								

**16) SC**	834	1212	1179	1216	1058	1516	1161	1466	1021	890	511	1166	910	676	615																							

**17) Alb**	349	838	844	677	514	1099	730	1316	622	366	441	993	613	749	618	341																						

**18) Gr**	380	904	1064	658	512	1104	782	1312	686	255	311	819	498	774	563	531	136																					

**19) Ma**	517	965	1135	792	713	1337	887	1323	783	440	266	841	592	667	500	222	144	138																				

**20) Ro**	570	1029	1221	833	745	1193	942	1476	819	502	409	828	620	889	715	198	274	341	180																			

**21) Tur**	159	447	700	265	413	696	460	1421	438	217	599	1008	622	899	891	845	352	303	490	535																		

**22) Irn**	494	424	717	369	727	805	601	1756	820	478	916	1134	813	1233	1285	1376	869	766	994	990	270																	

**23) Irs**	311	509	621	418	516	675	538	1528	587	566	860	1410	1042	874	896	991	529	592	781	773	217	370																

**24) Iq**	245	516	628	374	406	444	320	1422	265	510	970	1397	915	1127	1113	1051	557	550	754	859	270	541	315															

**25) Cy**	127	448	791	196	176	534	246	1241	240	395	1064	1239	799	1539	1359	1099	531	531	714	699	326	595	486	378														

**26) Sy**	152	464	637	398	322	421	336	1304	177	508	947	1429	941	1043	1045	911	481	487	655	712	197	562	277	114	329													

**27) Lb**	71	256	480	281	334	493	173	1330	191	426	925	1288	739	1213	1146	956	492	494	651	694	180	416	354	215	211	116												

**28) Jo**	183	513	704	373	451	489	141	139	123	561	1026	1296	840	1365	1247	988	577	661	758	758	410	765	578	246	266	255	204											

**29) SA**	448	580	605	606	724	565	372	1302	339	924	1286	1728	1256	1302	1357	1208	889	962	1115	1103	553	757	420	254	610	262	380	334										

**30) Qa**	647	819	805	973	948	696	454	1483	518	1196	1405	1769	1360	1506	1450	1351	1132	1216	1327	1225	903	1081	690	499	800	546	623	392	153									

**31) UA**	324	457	419	513	676	712	266	1304	367	818	1106	1575	1125	1233	1206	1050	671	825	956	954	488	694	365	290	500	305	315	295	130	249								

**32) Om**	477	626	625	651	745	765	417	1144	366	955	1223	1754	1313	1146	1227	1097	804	880	1001	1086	586	900	524	289	653	303	474	381	99	279	157							

**33) Ye**	769	913	1000	854	920	586	483	1438	383	1240	1664	1825	1407	1816	1734	1502	1252	1310	1412	1367	1088	1341	1066	542	768	645	710	369	365	238	475	410						

**34) Eg**	185	365	655	289	355	742	205	988	183	598	1128	1481	1068	1305	1285	1036	593	647	728	839	384	724	502	350	197	260	242	283	430	677	364	358	672					

**35) Mo**	764	999	1220	944	611	1264	801	933	715	1067	1543	1715	1365	1775	1652	1258	888	938	991	1125	1092	1492	1251	1098	559	970	911	903	1157	1282	1055	996	1105	454				

**36) Alg**	437	641	1001	456	502	958	425	1101	350	812	1325	1458	1240	1632	1487	1208	801	874	907	999	793	1120	909	697	289	654	578	487	783	895	643	676	735	215	272			

**37) Tun**	456	676	952	522	580	911	345	1153	316	893	1335	1485	1179	1653	1501	1250	853	919	968	1078	854	1193	969	604	332	626	554	356	643	664	528	551	497	251	379	71		

**38) EO**	1089	1203	1319	1207	1056	1659	1090	452	1021	1332	1648	1851	1582	1701	1686	1387	1134	1186	1191	1418	1330	1726	1442	1319	967	1187	1186	1231	1197	1394	1190	998	1319	651	323	569	666	

**39) EA**	826	932	1018	907	969	1107	622	555	569	1274	1653	1857	1497	1715	1692	1425	1170	1261	1290	1430	1154	1516	1172	819	790	803	844	676	617	693	677	500	581	449	638	473	397	346

*- For populations names see figure 4.

**Table 4:** mtDNA genetic distance matrix (×1000)*

1) EEJ	1	2	3	4	5	6	7	8	9	10	11	12	13	14	15	16	17	18	19	20	21	22	23	24	25	26	27	28	29	30
**2) IqJ**	916																													

**3) IqJ**	892	627																												

**4) MJ**	400	1020	814																											

**5) LJ**	1016	1303	770	741																										

**6) TnJ**	908	1336	973	438	487																									

**7) BJ**	453	817	676	381	727	605																								

**8) TrJ**	591	813	445	287	605	530	300																							

**9) YJ**	1020	1058	1257	1124	1349	1323	1287	1264																						

**10) EJ**	1685	1789	1794	1882	1701	1662	1844	1916	1251																					

**11) Pa**	417	976	941	674	1005	812	501	690	843	1382																				

**12) Tur**	531	478	419	499	767	795	406	379	985	1726	556																			

**13) Gr**	540	676	443	302	680	465	365	228	1138	1771	627	199																		

**14) It**	437	698	516	324	705	574	295	226	1247	1759	582	237	135																	

**15) Ge**	606	745	533	360	791	528	360	275	1299	1867	701	357	112	176																

**16) Fr**	504	836	646	334	814	590	379	316	1374	1880	710	379	173	126	93															

**17) Br**	610	761	562	341	822	602	454	295	1310	1927	806	410	166	220	70	84														

**18) Ru**	650	785	510	411	716	534	432	300	1355	1854	697	303	124	148	105	96	142													

**19) Po**	687	749	585	453	810	561	428	308	1414	1886	752	355	156	167	77	100	126	66												

**20) Sp**	557	778	680	370	843	657	445	339	1294	1719	712	368	251	181	214	167	207	184	206											

**21) Cy**	520	732	539	374	626	600	302	335	1141	1689	616	269	244	199	374	363	425	370	407	364										

**22) Lb**	543	736	618	502	729	633	390	456	1095	1520	383	233	348	288	485	463	554	425	482	412	270									

**23) Sy**	581	431	564	676	891	950	580	576	820	1465	463	283	427	444	613	659	659	609	659	609	412	339								

**24) In**	583	553	464	681	879	995	561	571	888	1697	576	209	422	369	568	579	613	513	576	543	397	425	341							

**25) Jo**	591	647	461	672	816	788	562	490	892	1329	419	387	449	370	613	616	711	563	614	532	355	328	285	405						

**26) SA**	631	731	799	863	964	1018	745	801	745	1123	478	579	679	668	836	875	841	849	898	805	567	561	416	503	486					

**27) Ye**	1064	1393	1351	1217	1310	1427	1206	1289	897	830	871	1078	1205	1154	1343	1315	1383	1314	1383	1254	1110	1125	949	943	898	770				

**28) Eg**	634	721	853	751	967	895	692	763	791	985	374	556	656	620	835	868	926	801	869	714	574	449	365	572	270	398	782			

**29) MoA**	736	1030	942	659	868	780	645	615	1196	1238	556	700	611	513	666	625	690	608	638	487	526	559	638	752	427	678	888	416		

**30) MoB**	674	948	851	568	880	728	595	511	1208	1386	550	626	494	415	504	450	507	470	486	348	499	535	595	701	442	679	1015	495	89	

**31) Et**	1394	1578	1679	1543	1492	1443	1541	1649	1008	300	1051	1470	1517	1470	1626	1612	1685	1604	1649	1461	1357	1279	1147	1406	1015	847	751	607	888	1032

*- For populations names see figure 5.

Correlations between genetic distance and geography and between genetic distance matrices based on different markers (excluding the non-Caucasoid populations Ethiopians and Ethiopian Jews) are shown in table 5. The autosomal polymorphisms have a very high correlation (0.789) with geography in contrast to the more moderate correlations of the X-chromosomal, Y-chromosomal and mtDNA polymorphisms (0.540, 0.395 and 0.641 respectively). In order to compare two competing theories regarding the origin of EEJ, their geographic distances were computed as if they originated from Italy or Israel, i.e. the great circle distances for EEJ were calculated not between Warsaw and other capitals, but between Rome or Jerusalem and other capitals. The correlation between the autosomal genetic distance matrix and geography was slightly higher, 0.804, for Rome but dropped to 0.694 for Jerusalem. Autosomal distances are much better correlated with mtDNA distances (0.826) and with X-chromosomal distances (0.732) than with Y-chromosomal distances (0.437). The correlations between the mtDNA and X-chromosomal matrices and the Y-chromosomal matrix are rather poor (0.206 and 0.241 respectively) and insignificant. When the correlations with geography were only calculated for the genetic distances of EEJ and not for the entire matrix (table 6), the same trends emerge with the autosomal correlation from Rome reaching a high of 0.926. The correlations from Jerusalem are negative for the autosomes, the X chromosome and mtDNA. The reverse is true for the Y chromosome.

**Table 5:** Correlation and significance level between genetic distance matrices and between genetic distance and geography

	Autosomes		Y		mtDNA		Geography	
	**r**	**p**	**r**	**p**	**r**	**p**	**r**	**p**

Autosomes*							0.789	0.0001

Y*	0.437	0.0021					0.395	0.0038

mtDNA*	0.826	0.0001	0.206	0.1200			0.641	0.0003

X**	0.732	0.0005	0.241	0.1399	0.633	0.0058	0.540	0.0022

* - Based on the 14 populations (excluding Ethiopian Jews) in the autosomal matrix

** - Based on the 10 populations (excluding Ethiopian Jews) common to all 4 matrices

r = correlation; p = significance level

**Table 6:** Correlation between the genetic distances of EEJ and geography*

	Warsaw	Rome	Jerusalem
Autosomes**	0.778	0.926****	-0.149

X***	0.781	0.835	-0.685

Y**	-0.613	-0.213	0.556

mtDNA**	0.471	0.779	-0.190

* - Great circle distances calculated from the three alternatives for their origin

** - Based on the 14 populations (excluding Ethiopian Jews) in the autosomal matrix

*** - Based on the 10 populations (excluding Ethiopian Jews) common to all 4 matrices

**** - When the Italians are removed, the correlation still remains very high, 0.904.

## Discussion

The autosomal genetic distance analysis presented here clearly demonstrates that the investigated Jewish populations do not share a common origin. The resemblance of EEJ to Italians and other European populations portrays them as an autochthonous European population. A study conducted in a New York college in the 1920s point to the same Ashkenazi - Italian similarity on basis of physical characteristics. Freshmen were asked before they knew one another to indicate the origin of their fellow students. Forty percent of the Italians were taken to be Ashkenazi Jews, and the same percentage of Ashkenazi Jews was adjudged Italians [24]. EEJ seem to be mainly Italian (Roman) in origin, which is easily understood, considering the historical evidence presented above.

The high correlation between the autosomal genetic distances and geography and the reduced correlation when EEJ are taken to originate from the Land of Israel reinforce the European origin of EEJ. In fact the correlation of the autosomal markers with geography is higher than previously described for 49 classical markers (0.503) or ~300,000 autosomal SNPs (0.661) in Europe [25]. If for comparison, only non-Jewish European populations are included, the correlation is lower, 0.689, but still higher than the above mentioned correlations. It is also interesting to note how using the three geographic alternatives for EEJ, changes the correlation, when only European populations are included. The correlation remains almost the same, 0.679, for Rome but drops to 0.490 and 0.571 for Warsaw and Jerusalem respectively; further emphasizing the correct geographic origin of EEJ within Europe.

### Biparental versus uniparental markers

At first sight it seems that there is more than one explanation for the differing results produced by the analysis of the NRY haplogroups. It thus seems possible that EEJ founder population in Rome was composed of exiled Israelite males and local Roman females. In its simple form this clearly contradicts the facts, because both the autosomal and X-chromosomal polymorphisms demonstrate that EEJ do not occupy an intermediate position between European and Middle Eastern populations, but rather a strict European one. From table 1 it is clear that Italians are as close or closer to the other Jewish populations and Palestinians as EEJ. It is possible that once the founder population was established no other males but many females joined it, thus creating a population that is almost entirely European in all genetic aspects apart from its Y chromosomes. Such phenomenon was described for the population of Antioquia, Columbia, where the autosomes point to 79% of European ancestry and only 16% of Amerindian ancestry, whereas according to mtDNA the ancestry is 90% Amerindian and only 2% European (there is also a small African component). Historical records demonstrate that local Amerindian females joined the population only at its beginning, whereas European males joined it also in later periods [26]. The suggestion that the proselyte ancestors of EEJ were almost entirely females does not however accord with what we know about conversion to Judaism [10,12,27-29].

The inference that the NRY points to a Middle Eastern origin of EEJ is erroneous not only because the Y chromosomal analysis contradicts the analyses based on the other chromosomes, and because the NRY is a single uniparental marker that does not represent the whole history of the population, but also because its smaller effective population size makes it much more vulnerable to severe genetic drift caused by demographic bottlenecks. The demographic histories of three Jewish populations exemplify how different demographic patterns make the uniparental markers more reliable for Iraqi (Babylonian) Jews and Yemenite Jews and less reliable for EEJ. Both Yemenite Jews and Iraqi Jews resemble populations from their regions of origin according to autosomal markers [1,3,30-32]. Yemenite Jews, who are usually considered a small isolate, were numerous enough to have an independent kingdom in the first centuries AD [33]. They numbered a few hundred thousand in the 12th century AD, and gradually declined; reaching only about 30-40,000 in the beginning of the 20th century [34]. Babylonian Jews numbered more than a million in the first century AD [35], and constituted the majority of the population in the area between the Euphrates and the Tigris in the 2^nd^-3^rd ^centuries AD [36]. Gilbert [37] estimates that by 600 AD there were 806,000 Jews in Mesopotamia, and according to Sassoon [38] it was inhabited by about a million Jews in the 7^th ^century. In the 14^th ^century the estimates for Baghdad alone range from 70,000 to hundreds thousands [38]. By 1939, 11 years before their emigration, there were 91,000 Jews in Iraq [35]. In contrast, the Jewish population of the Polish-Lithuanian Kingdom (EEJ) went through the opposite process. Their history is one of founder effects, migrations, demographic bottlenecks and finally a rapid expansion. We know nothing about their number in the first millennium, but after their emigration from Italy to Western Europe it is estimated that they numbered 4,000 in 1000 and 20,000 a hundred years later [8]. In 1500 already in Eastern Europe they numbered 10,000-30,000, in 1648 230,000-450,000 and in 1764 750,000 [39-41]. In the 19^th ^century because of the partitions of the Polish-Lithuanian Kingdom and the immigrations of Jews to Central and Western Europe and America, the estimation of the number of EEJ becomes more difficult, but there is no doubt that the increase in numbers was impressive, as the number of EEJ under Russian rule alone was 5,200,000 in 1897 [41].

**Figure 6:**
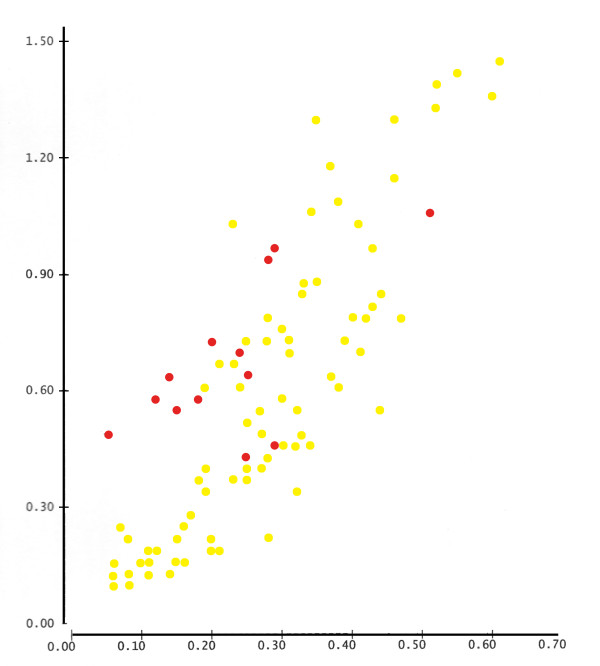
**Correlation of autosomal (X axis) and mtDNA (Y axis) distances**. Red circles denote EEJ. Most of the mtDNA distances of EEJ are too high relative to their autosomal distances, in contrast to most other distances (r = 0.826), attesting the greater genetic drift, to which the uniparental markers of EEJ were subjected.

The existence of severe demographic bottlenecks in the history of EEJ has also been suggested by genetic studies of disease-causing-mutations and mtDNA [42-46]. The comparison based on this second uniparental marker, mtDNA, may help to resolve from within genetics itself the problem of the Y chromosome reliability for inferring the origin of the male ancestors of EEJ. If the European and Middle Eastern contributions to the gene pool of EEJ were female and male respectively, then comparisons based on mtDNA must place EEJ among other European populations, distant from Middle Eastern populations. The mtDNA analysis presented in this study does not place EEJ among other European populations rather their position is more intermediate and marginal, as can be seen in figure 5 and in figure 6, where autosomal distances are correlated with mtDNA distances. This lends further support to the notion that because of the unique demographic history of EEJ, their uniparental markers were subjected to stronger genetic drift than the biparental markers and thus should not be used to trace their origin.

The data on the Y chromosome itself also support the unreliability of the uniparental markers for discovering the origin of EEJ. Nebel et al. [47] studied haplogroup R-M17, whose frequency is ~12% in Ashkenazi Jews. By comparing the structure of the STRs network among the various Ashkenazi populations and among the various European non-Jewish populations they reached the conclusion that a single male founder introduced this haplogroup into Ashkenazi Jews in the first millennium. Behar et al. [48] write "It is striking that whereas Ashkenazi populations are genetically more diverse at both the SNP and STR level compared with their European non-Jewish counterparts, they have greatly reduced within-haplogroup STR variability ... This contrasting pattern of diversity in Ashkenazi populations is evidence for a reduction in male effective population size, possibly resulting from a series of founder events and high rates of endogamy within Europe. This reduced effective population size may explain the high incidence of founder disease mutations despite overall high levels of NRY diversity". It is unlikely that EEJ are the descendants of a single population. Admixture coupled with small effective population size and bottlenecks can create the puzzling situation we encounter in the uniparental markers. Thus smaller contributions from several populations, including possibly the original Middle Eastern Jewish population, and a major contribution from Italy combined with the unique demography of EEJ can create the current genetic picture without the need to invoke a major contribution from the Middle East, which contradicts the autosomal and X-chromosomal data.

### Comments on previous studies

Some previous studies based on classical autosomal markers concluded that EEJ are a Middle Eastern population with genetic affinities to other Jewish populations. The problems with these studies have been previously discussed in detail [1]. These studies used fewer markers (mostly the less reliable antigenic markers) and failed to include European Mediterranean populations, apart from the discriminant analysis of Carmelli and Cavalli-Sforza [49], which used only four markers and contradicts the results of the later more elaborate discriminant analysis [1], and the genetic distance analysis of Livshits et al. [32], which includes a single European Mediterranean population, Spain. Despite this when a genetic distance analysis was performed, the greater similarity of EEJ to Russians and to a lesser extent to Germans more than to Non-European Jews was evident [32]. In fact Russians were more similar to EEJ than to any Non-Jewish European population in that analysis.

Recently, Cochran et al. [50] used 251 autosomal loci to calculate genetic distances and concluded that "from the perspective of a large collection of largely neutral genetic variation Ashkenazim are essentially European, not Middle Eastern". More recently, thousands of SNPs were used by Need et al. [51] to infer the relationships between Ashkenazi Jews and non-Jewish Europeans and Middle Easterners. They concluded that Ashkenazi Jews lie approximately midway between Europeans and the Middle Easterners, implying that Ashkenazi Jews may contain mixed ancestry from these two regions, and that they are close to the Adygei population from the Caucasus. However these conclusions are ill-founded, because, they used a highly selected set of SNPs, which were selected specifically for the purpose of distinguishing between Ashkenazi Jews and other populations and they inferred the origin of Ashkenazi Jews from principal components analysis (PCA), but as Tian et al. [52] show "PCA results are highly dependent on which population groups are included in the analysis. Thus, there should be some caution in interpreting these results and other results from similar analytic methods with respect to ascribing origins of particular ethnic groups'" Tian et al. [52] also published a table of paired Fst distances based on 10,500 random SNPs, which demonstrates that Ashkenazi Jews are not at all close to the Adygei population, and similarly to what is seen in table 1, their smallest distance is to Italians and then to Greeks. Unlike the assertion of Need et al. [51] on the midway position, and again similarly to what is seen in table 1, Italians and Greeks are closer to the Middle Eastern populations than Ashkenazi Jews.

The same phenomenon is seen in the table of Fst distances of Atzmon et al. [53]. North Italians (Bergamo and Tuscany) are a little closer to the Jewish and Middle Eastern populations than Ashkenazi Jews. The Italians from Tuscany (surprisingly the sample from Bergamo was not used) in Behar et al. [54] are also closer to the Jewish and Middle Eastern populations than Ashkenazi Jews. The Italians from Tuscany are in fact the closest population to Ashkenazi Jews in Behar et al. [54]. There is one sample that is apparently a little closer, what they call Sephardic Jews. Unfortunately this sample is composed of two populations, Turkish Jews and Bulgarian Jews, which should have been studied separately like all other Jewish populations. Bulgarian Jews have been shown in the past based on autosomal classical markers to be closer to EEJ than to populations with Sephardic ancestry and considering their history it was concluded that the Ashkenazi component in their gene pool is at least as large or even larger that the Sephardic component [1]. From both The current study and those of Atzmon et al. [53] and Behar et al. [54] it can be seen that the only Jewish populations that are as close to Ashkenazi Jews as non-Jewish Europeans are those with a significant Sephardic (The descendants of the Jews who were expelled from the Iberian peninsula at the end of the 15^th ^century) component in their gene pool. It is not possible at this stage to say what is the source of this resemblance, since we don't know what is the origin of Sephardic Jews, but considering all the genetic affinities of both groups it likely stems from Sephardic Jews being the descendants of converts in the Mediterranean basin rather than from a common Jewish origin in the Land of Israel. When one compares the autosomal distances of EEJ (current study) or Ashkenazi Jews (in Atzmon et al. [53] and Behar et al. [54]) from the Jewish populations that were investigated in the current study, Iraqi, Iranian, Moroccan, Yemenite and Ethiopian Jews, one finds perfect agreement. EEJ or Ashkenazi Jews are much closer to non-Jewish Europeans than to these Jewish populations in all three studies.

The studies of Atzmon et al. [53] and Behar et al. [54] are based on 164,894 and 226,839 SNPs respectively. While this impressive number reduces the errors of the distances that stem from the number of markers, the errors that stem from sampling only a small number of individuals are much larger in these studies, where sample sizes can be as small as 2-4 individuals. The effect of these errors can be seen in table 7. Despite the small number of markers the current matrix has the highest correlation with geography. Moreover it has a higher correlation with each of the two other matrices than the two of them have with each other. The high correlations between the current matrix and the other two attest for the robustness of the autosomal genetic distances in this study. The lower correlation between the two matrices, which are based on more than 150,000 SNPs, is surprising and even more so, if we remember that the four non-Jewish populations are represented by exactly the same individuals taken from the Human Genome Diversity Panel (HGDP). It is likely then that sampling more individuals, which represent more of the variation of the investigated populations, is far more important than typing many markers. It is also possible that the typing error rates of genome-wide microarray studies are much higher, as demonstrated by the genotyping errors that were discovered in 7 out of 29 (24%) reexamined SNPs [55]. It seems therefore, that good characterization of the genetic relationships between populations can be achieved by a small number of good unique-event-polymorphisms.

**Table 7:** Comparison of the correlations of the three autosomal genetic distance matrices*

	Current Study		Atzmon et al.		Geography**	
	**r**	**p**	**r**	**p**	**r**	**p**

Current Study					0.561	0.0015

Atzmon et al. 2010	0.872	0.0003			0.482***	0.0192

Behar et al. 2010	0.852	0.0012	0.788	0.0029	0.437****	0.0351

* - Based on the 7 populations common to all 3 studies

** - Great circle distances for EEJ or Ashkenazi Jews calculated from Rome (in all cases this was the highest correlation)

*** - Great circle distances for Italians calculated from Parma

**** - Great circle distances for Italians calculated from Florence

r = correlation; p = significance level

## Conclusions

EEJ are Europeans probably of Roman descent who converted to Judaism at times, when Judaism was the first monotheistic religion that spread in the ancient world. Any other theory about their origin is not supported by the genetic data. Future studies will have to address their genetic affinities to various Italian populations and examine the possibility of other components both European and Non-European in their gene pool.

## Competing interests

The author declares that he has no competing interests.

## Reviewers' comments

### Reviewer's report 1

Damian Labuda, Pediatrics Department, Montreal University Sainte-Justine Hospital Research Center, Montreal, PQ Canada (nominated by Jerzy Jurka, Genetic Information Research Institute, Mountain View, California USA).

The author compiled and reanalyzed the data on autosomal and sex chromosomes polymorphisms collected by different laboratories on different Jewish and West-Eurasiatic populations. His analysis indicates much greater European component of Eastern European Jews, EEJ (essentially Ashkenazim) than of other Jewish groups. Moreover the analysis points to Italians as the closest population to EEJ.

The question is how to interpret this evidence. Imperial Rome was a very cosmopolitan city culturally and genetically diverse. To what extent a sample of contemporary Italians preserves the genetic link to its population? It can simply reflect a mixture of historical influences from different centers around the Mediterranean Sea. We should thus keep in mind that the Italian connection may simply indicate Southern European and Mediterranean links with the latter including Middle Eastern roots.

Interestingly, this analysis that is based on a limited number of markers provided results that are very similar to a paper of Atzmon and colleagues, published five days ago in the American Journal of Human Genetics, and based on the microarray-based genotyping genome of wide distributed markers. I would like the author to comment on this paper in the context of his findings and his thoughts and reflections on the origin of Jewish Diasporas. Should we go back to the single locus analyses, as in the case of uniparentally transmitted markers, but targeting one by one different individual segments of the nuclear genome? Perhaps, in this way we could partition and identify genetic ancestries of different populations, which due to their history of relative isolation, are considered as genetically homogenous.

The author refers to Sangvi's G2 as the most appropriate distance metrics. Could you make it more clear when this metric was used and when that of Reynolds (only to produce a tree?).

#### Author's response

The historical sources listed above show that conversion to Judaism was common in ancient Rome among all ranks of the Roman society including the imperial families. It is thus unlikely that the original Roman population did not constitute a significant portion of the proselytes. What else can explain the resemblance of EEJ to a general sample of Italians in this study and to more local samples in the two array studies [53,54]? In all three studies the genetic affinities of the Ashkenazim are very similar to the affinities of the Italians, with the Ashkenazim usually being a bit more distant from the other populations, as can be expected from a population that underwent a stronger genetic drift. It is thus unlikely that the Ashkenazim are a mixture of people from different places in the Mediterranean basin, unless current-day Italians themselves not only have absorbed foreign genetic contributions, but actually constitute such a mixture, and this seems unlikely as well. The very high correlation (0.926) between the genetic distances of EEJ and geographic distances, when the latter are calculated from Rome, also supports the origin of EEJ from Italy or its vicinity and not merely from the Mediterranean basin. The similarity to Italians was also evident when several Italian populations from different provinces were included in a comparison based on classical autosomal markers. Most Italian populations were closer to EEJ than all other populations (data not shown).

My comments on the papers by Atzmon et al. [53] and Behar et al. [54] are in the discussion. Studying autosomal haplotypes will indeed contribute to revealing the ancestries of populations, but in order to gain meaningful insights one ought to study at least several loci and ensure that sample sizes are adequate, this may entail more effort than studying single SNPs, and I am not sure that the affinities between the populations are going to be depicted more accurately. I changed the phrasing in Methods to make it clearer that the formula of Reynolds et al. was only used for the calculation of the tree.

### Reviewer's report 2

Kateryna Makova, Department of Biology, Penn State University, Pennsylvania USA.

This is an interesting manuscript that presents intriguing results. I have only a few comments:

1. The introduction is very short, while the discussion is lengthy. I suggest moving parts of the Discussion to the Introduction.

2. Some of the statements in the Discussion are too strong. I disagree with statements about "erroneous Y chromosomal genetic distances", "both uniparental markers should not be used to trace their origin", "uniparental markers being unreliable". The author should modify them.

#### Author's response

I moved the paragraph on the history of EEJ to the Introduction. The current revised version of the paper includes a new comparison based on mtDNA. I maintain that it adds more weight to my assertion that the uniparental markers should not be used to trace the origin of EEJ. In no way did I mean that the uniparental markers are always unreliable; to clarify it I modified the relevant sentence in the discussion. Indeed from the demographic examples that I give in the Discussion, it seems that the uniparental markers can be used to study the origins of Iraqi Jews and Yemenite Jews.

### Reviewer's report 3

Qasim Ayub, The Wellcome Trust Sanger Institute, Wellcome Trust Genome Campus, Hinxton, UK (nominated by Dan Graur, Department of Biology and Biochemistry, University of Houston, Houston, USA).

The paper by Zoossmann-Diskin entitled 'The origin of Eastern European Jews revealed by autosomal and sex chromosomal polymorphisms' explores autosomal and sex chromosomal polymorphisms in six Jewish populations using previously published and additional unpublished data. The author concludes that the Jewish populations examined do not share a common origin and that Eastern European Jews are closer to the Italian population.

My major concern is the choice of markers and populations used in this study. The author has analyzed 17 autosomal loci, including 9 polymorphic protein electrophoretic variants in which the genotype was assumed. Although phenotypes often do correlate with genotypes assuming that they do can lead to erroneous results. Of the remaining 8 it is unclear whether the same samples were genotyped as the sample numbers for each locus vary widely (Supplementary Tables 2-4).

The author also uses Y hapologroup frequencies and shows a multidimensional scaling plot of Y chromosomal genetic distance matrix. However, the supplementary data (Supplementary Table 5) lists an outdated nomenclature for Y haplogroups as the M78 marker is no longer considered part of haplogroup E3b1. It would be more appropriate to list which markers are used to designate the haplogroups to ensure that they are comparable. In addition, the haplogroups that are selected for these analyses do not provide phylogenetic resolution to reliably detect male genetic sub-structure within the Middle East. The omission of recent mtDNA studies (Behar et al., 2008, PLoS One 3:e2062) is surprising as is the use of a single X chromosomal locus (DYS44) to make broad conclusions about genetic relatedness.

Current evidence, supported more recently by two major studies carried out on Jewish populations (Atzmon et al., Am J H Genetics 86:850-859; Behar et al., Nature doi:10.1038) using a much larger dataset clearly demonstrate a common genetic thread linking the diverse Mizrahi, Sephardic and Ashkenazi Jewish populations with the populations from the Levant and Middle East. The Ashkenazi show a European component but this is shared with many Eastern and Southern Europeans populations. These studies contradict the author's conclusion and demonstrate the power of using unbiased markers and host populations in corresponding geographic regions to address issues such as genetic relatedness among Jewish and non-Jewish populations

#### Author's response

I am not sure what Dr Ayub means by "assumed", but I suspect that he means something like the relationships between phenotype and genotype in certain blood groups, in which one (or more) allele is dominant over the other and the gene frequencies of the alleles have to be inferred from the phenotypes assuming Hardy-Weinberg equilibrium. In such cases there may indeed be errors in the gene frequencies. Protein electrophoretic markers are completely different. Nothing is inferred! As mentioned in Methods all the protein electrophoretic markers in this study represent a SNP at the DNA level. This SNP causes an amino acid change that can be detected at the protein level. Both alleles are directly viewed on the gel in the same way as both alleles of an RFLP are directly viewed on the gel. Gene frequencies are determined in both cases by simple gene counting and the error rate in protein electrophoresis is no greater than in DNA studies. There is no need to type the same samples for all the polymorphisms, because the unit of study is the population, not the individual. One can use polymorphisms typed by different researchers using different samples and combine them to create a genetic profile of each population. Typing all the polymorphisms on the same sample does not add more credibility to the study. Indeed the renowned works that employed classical autosomal markers to portray the genetic affinities of human populations were based on many different samples typed by many different researchers [56,57].

The nomenclature in the Y chromosome supplementary table has been updated. Following the publication of the study by Behar et al. [54] it was possible to add more Jewish populations to the Y chromosome analysis and increase the number of chromosomes for the Jewish populations. This increase has come however at the expense of resolution, because Behar et al. [54] used fewer haplogroups in their analysis. Consequently the number of haplogroups was reduced from 15 in the original version to 14 in this revised version. I would have been happier if the available data on the Jewish populations had enabled greater resolution to reliably detect male genetic sub-structure within the Middle East, but since this work deals with the genetic affinities of EEJ, the current level is sufficient. The work of Behar et al. from 2008 was instrumental in creating the mtDNA matrix as can be seen in table 7 in Additional file 1. There was no need to cite it previously, as it did not contain any genetic distance analysis that could further clarify the origin of EEJ. I am surprised at Dr Ayub's surprise at the use of a single X chromosomal locus. It would have been better to use many X chromosomal loci, but even the use of single loci is advantageous, as I am sure even Dr Ayub would agree regarding the two other single loci that I use, the non-recombining Y chromosome (NRY) and mtDNA.

As written in the Discussion the genetic distance matrices of Atzmon et al. [53] and Behar et al. [54] do not contradict my results, but reinforce them. I completely reject Dr Ayub's claim that the markers or populations I used are biased in anyway, and I let the reader judge, where exactly the bias lies.

## Supplementary Material

Additional file 1 **Allele frequencies tables, Tables S1-S7**. The file contains seven tables that give the allele frequencies of the employed polymorphisms. Click here for file

## References

[B1] Zoossmann-Diskin A Joel A Liron M Kerem B Shohat M Peleg L Protein electrophoretic markers in Israel: compilation of data and genetic affinities Ann Hum Biol 2002 29 142 175 10.1080/03014460110058971 11874621

[B2] Bonné-Tamir B Zoossmann-Diskin A Teicher A Oppenheim A Nevo S Roberts DF, Fujiki N, Torizuka K Genetic distance analyses in Israeli groups using classical markers and DNA polymorphisms in the β-globin gene Isolation migration and health 1992 Cambridge: Cambridge University Press 87 106

[B3] Zoossmann-Diskin A Gazit E Peleg L Shohat M Turner D Thrombophilic polymorphisms in Israel Blood Cells Mol Dis 2008 41 230 233 10.1016/j.bcmd.2008.05.004 18583164

[B4] Ankory Z Goodman RM, Motulsky AG Origins and history of Ashkenazi Jewry (8th to 18th century) Genetic diseases among Ashkenazi Jews 1979 New York: Raven Press 19 46

[B5] Hendelssmann Y Shavit S, Shamir I The Jewish centre in Ashkenaz in the 10-13th centuries The History of the people of Israel 1985 Givataim, Israel: Massada 72 73 (in Hebrew)

[B6] Renan E Le judaÏsme comme race et comme religion 1883 Paris: Ancienne Maison Michel Lévy Frères

[B7] Ashdy L Knaani D Germany, Jews Encyclopedia of social sciences 1970 1 Tel-Aviv: Sifriat-Poalim 849 862 (in Hebrew)

[B8] Kriegel M Barnavi E The beginnings of European Jewry, 500-1096 A historical atlas of the Jewish people 1992 London: Hutchinson 78 79

[B9] Halpern I Finkelstein L The Jews in Eastern Europe (From Ancient Times until the Partitions of Poland, 1772-1795) The Jews, their history, culture and religion 1960 1 New York: Harper & Brothers Publishers 287 319

[B10] Rapaport U Jewish religious propaganda and proselytism in the period of the second commonwealth PhD thesis 1965 The Hebrew University (in Hebrew with an English abstract)

[B11] Eilat T The Jewish community in pagan Rome from its beginning in times of the republic till Christianity became a recognized religion of the Roman Empire MA thesis 1979 Tel-Aviv University (in Hebrew with an English abstract)

[B12] Feldman LH Stern M Proselytism and syncretism The Diaspora in the Hellenistic-Roman world 1983 The Society For The Publication Of The History Of The Jewish People, Israel 188 207 (in Hebrew)

[B13] Meroz N Proselytism in the Roman Empire in the first centuries AD MA thesis 1992 Tel-Aviv University (in Hebrew with an English abstract)

[B14] Zoossmann-Diskin A Swinburne S Shohat M Peleg L Gazit E Turner D Typing classical polymorphisms by real-time PCR: analysis of the GPT and ALAD protein polymorphisms in the Jewish populations Am J Hum Biol 2008 20 490 492 10.1002/ajhb.20766 18432999

[B15] Wainwright BJ Scambler PJ Schmidtke J Watson EA Law HY Farrall M Cooke HJ Eiberg H Williamson R Localization of cystic fibrosis locus to human chromosome 7cen-q22 Nature 1985 318 384 385 10.1038/318384a0 2999612

[B16] White R Woodward S Leppert M O'connell P Hoff M Herbst J Lalouel JM Dean M Vande Woude G A closely linked genetic marker for cystic fibrosis Nature 1985 318 382 384 10.1038/318382a0 3906407

[B17] Zoossmann-Diskin A Gazit E Peleg L Shohat M Turner D 844ins68 in the cystathionine beta-synthase gene in Israel and review of its distribution in the world Anthropol Anz 2004 62 147 155 15228193

[B18] Rigat B Hubert C Corvol P Soubrier F PCR detection of the insertion/deletion polymorphism of the human angiotensin converting enzyme gene (DCP1) (dipeptidyl carboxypeptidase 1) Nucleic Acids Res 1992 20 1433 10.1093/nar/20.6.1433-a 1313972 PMC312213

[B19] Goltsov AA Eisensmith RC Woo SL Detection of the XmnI RFLP at the human PAH locus by PCR Nucleic Acids Res 1992 20 927 10.1093/nar/20.4.927-a 1347420 PMC312059

[B20] Jorde LB Mielke JH, Crawford MH The genetic structure of subdivided human populations Current developments in anthropological genetics 1980 1 New York: Plenum Press 135 208

[B21] Sanghvi LD Comparison of genetical and morphological methods for a study of biological differences Am J Phys Anthropol 1953 11 385 404 10.1002/ajpa.1330110313 13104652

[B22] Nei M Genetic distance between populations American Naturalist 1972 106 283 292 10.1086/282771

[B23] Reynolds J Weir BS Cockerham CC Estimation of the Coancestry Coefficient: Basis for a Short-Term Genetic Distance Genetics 1983 105 767 779 17246175 10.1093/genetics/105.3.767 PMC1202185

[B24] Herskovits MJ Finkelstein L Who are the Jews? The Jews, their history, culture and religion 1960 2 New York: Harper & Brothers Publishers 1489 1507

[B25] Lao O Lu TT Nothnagel M Junge O Freitag-Wolf S Caliebe A Balascakova M Bertranpetit J Bindoff LA Comas D Holmlund G Kouvatsi A Macek M Mollet I Parson W Palo J Ploski R Sajantila A Tagliabracci A Gether U Werge T Rivadeneira F Hofman A Uitterlinden AG Gieger C Wichmann HE Ruther A Schreiber S Becker C Nurnberg P Nelson MR Krawczak M Kayser M Correlation between genetic and geographic structure in Europe Curr Biol 2008 18 1241 1248 10.1016/j.cub.2008.07.049 18691889

[B26] Bedoya G Montoya P Garcia J Soto I Bourgeois S Carvajal L Labuda D Alvarez V Ospina J Hedrick PW Ruiz-Linares A Admixture dynamics in Hispanics: a shift in the nuclear genetic ancestry of a South American population isolate Proc Natl Acad Sci USA 2006 103 7234 7239 10.1073/pnas.0508716103 16648268 PMC1464326

[B27] Braude WG Jewish proselyting in the first five centuries of the common era, the age of the Tannaim and Amoraim 1940 Menasha, WI: The George Banta Publishing Company

[B28] Bamberger BJ Proselytism in the Talmudic period 1968 New York: Ktav Publishing House

[B29] Rosenbloom JR Conversion to Judaism: from the biblical period to the present 1978 Cincinnati: Hebrew Union College Press

[B30] Bonné-Tamir B Ahuja YN, Neel JV Oriental Jewish communities and their genetic relationships with South-West Asian populations Genetic Microdifferentiation in Human and other Animal Populations: Occasional Papers in Anthropology No. 1 1985 Delhi: Indian Anthropological Association 153 170

[B31] Bonné-Tamir B Zoossmann-Diskin A Teicher A Bonné-Tamir B, Adam A Genetic diversity among Jews reexamined: preliminary analyses at the DNA level Genetic diversity among Jews: Diseases and markers at the DNA level 1992 New York: Oxford University Press 80 94

[B32] Livshits G Sokal RR Kobyliansky E Genetic affinities of Jewish populations Am J Hum Genet 1991 49 131 146 2063865 PMC1683231

[B33] Ben-Zeev I The Jews in Arabia 1970 Jerusalem: Achiassaf (in Hebrew)

[B34] Ashdy L Knaani D Yemen, Jews Encyclopedia of social sciences 1970 5 Tel-Aviv: Sifriat-Poalim 853 854 (in Hebrew)

[B35] Baron SW Roth C, Wigoder G Population Encyclopaedia Judaica 1971 13 Jerusalem: Keter Publishing House 866 903

[B36] Grets T History of the Jews 1955 Hebrew Tel-Aviv: Izreel Publishing House

[B37] Gilbert M Jewish history atlas 1980 Hebrew Jerusalem: Edanim Publishers (By arrangement with Weidenfeld and Nicolson, London)

[B38] Sassoon DS A history of the Jews in Baghdad 1949 Letchworth, Great Britain

[B39] Baron SW Poland-Lithuania 1500-1650 A social and religious history of the Jews 1976 XVI New York: Columbia University Press

[B40] Polonsky A Polonsky A, Basista J, Link-Lenczowski A Introduction The Jews in old Poland, 1000-1795 1993 London: IB Tauris & Co Publishers 1 9

[B41] Stampfer S Bartal I, Gutman I Population growth and emmigration of the Polish-Lithuanian Jewry in the 16-19th centuries The broken chain, Polish Jewry through the ages 1997 Jerusalem: The Zalman Shazar Center 263 285 (in Hebrew)

[B42] Diaz GA Gelb BD Risch N Nygaard TG Frisch A Cohen IJ Miranda CS Amaral O Maire I Poenaru L Caillaud C Weizberg M Mistry P Desnick RJ Gaucher disease: the origins of the Ashkenazi Jewish N370S and 84GG acid beta-glucosidase mutations Am J Hum Genet 2000 66 1821 1832 10.1086/302946 10777718 PMC1378046

[B43] Frisch A Colombo R Michaelovsky E Karpati M Goldman B Peleg L Origin and spread of the 1278insTATC mutation causing Tay-Sachs disease in Ashkenazi Jews: genetic drift as a robust and parsimonious hypothesis Hum Genet 2004 114 366 376 10.1007/s00439-003-1072-8 14727180

[B44] Risch N Tang H Katzenstein H Ekstein J Geographic distribution of disease mutations in the Ashkenazi Jewish population supports genetic drift over selection Am J Hum Genet 2003 72 812 822 10.1086/373882 12612865 PMC1180346

[B45] Slatkin M A population-genetic test of founder effects and implications for Ashkenazi Jewish diseases Am J Hum Genet 2004 75 282 293 10.1086/423146 15208782 PMC1216062

[B46] Behar DM Hammer MF Garrigan D Villems R Bonné-Tamir B Richards M Gurwitz D Rosengarten D Kaplan M Della Pergola S Quintana-Murci L Skorecki K MtDNA evidence for a genetic bottleneck in the early history of the Ashkenazi Jewish population Eur J Hum Genet 2004 12 355 364 10.1038/sj.ejhg.5201156 14722586

[B47] Nebel A Filon D Faerman M Soodyall H Oppenheim A Y chromosome evidence for a founder effect in Ashkenazi Jews Eur J Hum Genet 2005 13 388 391 10.1038/sj.ejhg.5201319 15523495

[B48] Behar DM Garrigan D Kaplan ME Mobasher Z Rosengarten D Karafet TM Quintana-Murci L Ostrer H Skorecki K Hammer MF Contrasting patterns of Y chromosome variation in Ashkenazi Jewish and host non-Jewish European populations Hum Genet 2004 114 354 365 10.1007/s00439-003-1073-7 14740294

[B49] Carmelli D Cavalli-Sforza LL The genetic origin of the Jews: a multivariate approach Hum Biol 1979 51 41 61 422163

[B50] Cochran G Hardy J Harpending H Natural history of ashkenazi intelligence J Biosoc Sci 2006 38 659 693 10.1017/S0021932005027069 16867211

[B51] Need AC Kasperaviciute D Cirulli ET Goldstein DB A genome-wide genetic signature of Jewish ancestry perfectly separates individuals with and without full Jewish ancestry in a large random sample of European Americans Genome Biol 2009 10 R7 10.1186/gb-2009-10-1-r7 19161619 PMC2687795

[B52] Tian C Kosoy R Nassir R Lee A Villoslada P Klareskog L Hammarstrom L Garchon HJ Pulver AE Ransom M Gregersen PK Seldin MF European Population Genetic Substructure: Further Definition of Ancestry Informative Markers for Distinguishing Among Diverse European Ethnic Groups Mol Med 2009 15 371 383 10.2119/molmed.2009.00094 19707526 PMC2730349

[B53] Atzmon G Hao L Pe'er I Velez C Pearlman A Palamara PF Morrow B Friedman E Oddoux C Burns E Ostrer H Abraham's children in the genome era: major Jewish diaspora populations comprise distinct genetic clusters with shared Middle Eastern Ancestry Am J Hum Genet 2010 86 850 859 10.1016/j.ajhg.2010.04.015 20560205 PMC3032072

[B54] Behar DM Yunusbayev B Metspalu M Metspalu E Rosset S Parik J Rootsi S Chaubey G Kutuev I Yudkovsky G Khusnutdinova EK Balanovsky O Semino O Pereira L Comas D Gurwitz D Bonne-Tamir B Parfitt T Hammer MF Skorecki K Villems R The genome-wide structure of the Jewish people Nature 2010 466 238 242 10.1038/nature09103 20531471

[B55] Xue Y Zhang X Huang N Daly A Gillson CJ Macarthur DG Yngvadottir B Nica AC Woodwark C Chen Y Conrad DF Ayub Q Mehdi SQ Li P Tyler-Smith C Population differentiation as an indicator of recent positive selection in humans: an empirical evaluation Genetics 2009 183 1065 1077 10.1534/genetics.109.107722 19737746 PMC2778960

[B56] Cavalli-Sforza LL Piazza A Human genomic diversity in Europe: a summary of recent research and prospects for the future Eur J Hum Genet 1993 1 3 18 7520820 10.1159/000472383

[B57] Cavalli-Sforza LL Menozzi P Piazza A The history and geography of human genes 1994 Princeton: Princeton University Press

[B58] Weber W Untersuchungen von in Deutschland lebenden Türken in 23 Blutgruppensystemen; Bedeutung für die Biostatistik Das Ärztliche Laboratorium 1983 29 171 178

[B59] Walter H Gölge M Aksoy M Bermik E Sivasli A Genetic serum protein markers (HP, GC, TF, PI) in four Turkish population samples Int J Anthropol 1992 7 27 32 10.1007/BF02447867

[B60] Brega A Scacchi R Cuccia M Kirdar B Peloso G Corbo RM Study of 15 protein polymorphisms in a sample of the Turkish population Hum Biol 1998 70 715 728 9686482

[B61] Tsiakalos G Walter H Hilling M Schaarschmidt L Investigations on the distribution of genetic polymorphisms in Greece. 2. Serum protein polymorphisms Anthropol Anz 1981 39 1 9 6895292

[B62] Tsiakalos G Walter H Hilling M Investigation on the distribution of genetic polymorphisms in Greece. 3. Red cell enzyme polymorphisms and genetic distances Anthropol Anz 1981 39 244 254 6459757

[B63] Kouvatsi A Triantaphyllidis CD Gc and Tf subtypes in Greece Hum hered 1987 37 62 64 10.1159/000153679 3557464

[B64] Hundrieser J Bremer S Peinemann F Stuhrmann M Hoffknecht N Wulf B Schmidtke J Reiss J Maass G Tümmler B Frequency of the F508 deletion in the CFTR gene in Turkish cystic fibrosis patients Hum Genet 1990 85 409 410 10.1007/BF02428283 2210752

[B65] Devoto M De Benedetti L Seia M Piceni Sereni L Ferrari M Bonduelle ML Malfroot A Lissens W Balassopoulou A Adam G Loukopoulos D Cochaux p Vassart G Szibor R Hein J Grade K Berger W Wainwright B Romeo G Haplotypes in cystic fibrosis patients with or without pancreatic insufficiency from four European populations Genomics 1989 5 894 898 10.1016/0888-7543(89)90131-6 2574150

[B66] Weber J Aulehla-Scholz C Kaiser R Eigel A Neugebauer M Horst J Olek K Cystic fibrosis: typing 89 German families with linked DNA probes Hum Genet 1988 81 54 56 10.1007/BF00283729 2904405

[B67] Tummler B Aschendorff A Darnedde T Fryburg K Maass G Hundrieser J Marker haplotype association with growth in German cystic fibrosis patients Hum Genet 1990 84 267 273 10.1007/BF00200573 1968035

[B68] Schmidtke J Krawczak M Schwartz M Alkan M Bonduelle M Bühler E Chemke M Darnedde T Domagk J Engel W Frey D Fryborg K Halley D Hundrieser J Ladanyi L Libaers I Lissens W Mächler M Malik NJ Morreau J Neubauer V Oostra B Pape B Poncin JE Schinzel A Simon P Trefz FK Tümmler B Vassart G Voss R Linkage relationships and allelic associations of the cystic fibrosis locus and four marker loci Hum Genet 1987 76 337 343 10.1007/BF00272441 2886421

[B69] Dean M Amos JA Lynch J Romeo G Devoto M Ward K Halley D Oostra B Ferrari M Russo S Weir BS Finn PB Collins FS Iannuzzi MC Prenatal diagnosis and linkage disequilibrium with cystic fibrosis for markers surrounding D7S8 Hum Genet 1990 85 275 278 10.1007/BF00206745 1975555

[B70] Beaudet A Bowcock A Buchwald M Cavalli-Sforza L Farrall M King MC Klinger K Lalouel JM Lathrop G Naylor S Ott J Tsui LC Wainwright B Watkins P White R Williamson R Linkage of cystic fibrosis to two tightly linked DNA markers: joint report from a collaborative study Am J Hum Genet 1986 39 681 693 3026171 PMC1684135

[B71] Ramsay M Williamson R Estivill X Wainwright BJ Ho MF Halford S Kere J Savilahti E de la Chapelle A Schwartz M Super M Farndon P Harding C Meredith L Al-Jader L Ferec C Claustres M Casais T Nunes V Gasparini P Savoia A Pignatti PP Novelli G Bennarelli M Dallapicola B Kalaydjieva L Scambler PJ: Haplotype analysis to determine the position of a mutation among closely linked DNA markers Hum Mol Genet 1993 2 1007 1014 10.1093/hmg/2.7.1007 8364537

[B72] Harris A Beards F Mathew C Mutation analysis at the cystic fibrosis locus in the British population Hum Genet 1990 85 408 409 10.1007/BF02428282 2210751

[B73] McIntosh I Curtis A Lorenzo ML Keston M Gilfillan AJ Morris G Brock DJ The haplotype distribution of the delta F508 mutation in cystic fibrosis families in Scotland Hum Genet 1990 85 419 420 10.1007/BF02428290 2210756

[B74] Mornet E Simon-Bouy B Serre JL Estivill X Farrall M Williamson R Boue J Boue A Genetic differences between cystic fibrosis with and without meconium ileus Lancet 1988 1 376 378 10.1016/S0140-6736(88)91180-4 2893188

[B75] Vidaud M Kitzis A Ferec C Bozon D Dumur V Giraud G David F Pascal O Auvinet M Morel Y Andre J Chomel JC Saleun JP Farriaux JP Roussel P Labbé A Dastugue B Lucotte G Monnier N Foucaud P goossens M Feingold J Kaplan JC Confirmation of linkage disequilibrium between haplotype B (XV-2c, allele 1;KM-19, allele 2) and cystic fibrosis allele in the French population Hum Genet 1989 81 183 184 10.1007/BF00293899 2563252

[B76] Serre JL Simon-Bouy B Mornet E Jaume-Roig B Balassopoulou A Schwartz M Taillandier A Boué J Boué A Studies of RFLP closely linked to the cystic fibrosis locus throughout Europe lead to new considerations in populations genetics Hum Genet 1990 84 449 454 10.1007/BF00195818 1969843

[B77] Ferec C Verlingue C Parent P Desequilibre de liaison et marqueurs de l'ADN associes au gene de la fibrose kystique J Genet Hum 1989 37 407 423 2635718

[B78] Baranov VS Ivashchenko TE Gorbunova VN Voronina OV Gaĭtskhoki VS Gol'tsov AA Kaboev OK Shvarts EI Berlin YuA Livshits LA Buzhievskaya TI Venozhinskis MT Sokolov BP Kalinin VN Orlov AV Romanenko OP Luk'yanenko AN Kapranov NI Rachinskii SV Allele polymorphism of the DNA loci MET, D7S8, D7S23, linked to the cystic fibrosis gene in some populations of the USSR, in high risk families and in cystic fibrosis patients Genetika 1991 27 113 121 (in Russian) 2037248

[B79] Voronina OV Baranov VS Gaitskhoki VS Gorbunova VN Ivashchenko TE Shvartsman AL Polymorphism of nucleotide sequences of human genomic DNA linked to a mucoviscidosis locus Mol Gen Mikrobiol Virusol 1990 14 17 (in Russian) 1973261

[B80] Bal J Maciejko D Buława E Mazurczak T Zastosowanie sond molekularnych DNA w diagnostyce mukowiscydozy--analiza polimorfizmu dlugosci fragmentow restrykcyjnych (RFLP) w 22 rodzinach wysokiego ryzyka Pol Tyg Lek 1992 47 215 218 1359513

[B81] Akar N Akar E Misirlioğlu M Avcu F Yalçin A Cin S Search for genetic factors favoring thrombosis in Turkish population Thromb Res 1998 92 79 82 10.1016/S0049-3848(98)00113-3 9792115

[B82] Akar N Misirlioğlu M Akar E Avcu F Yalçin A Sözüöz A Prothrombin gene 20210 G-A mutation in the Turkish population Am J Hematol 1998 58 249 10.1002/(SICI)1096-8652(199807)58:3<249::AID-AJH20>3.0.CO;2-3 9662283

[B83] Balta G Gürgey A Methylenetetrahydrofolate reductase (MTHFR) C677T mutation in Turkish patients with thrombosis Turk J Pediatr 1999 41 197 199 10770658

[B84] Gürgey A Mesci L The prevalence of factor V Leiden (1691 G-- > A) mutation in Turkey Turk J Pediatr 1997 39 313 315 9339109

[B85] Ozbek U Tangün Y Frequency of factor V Leiden (Arg506Gln) in Turkey Br J Haematol 1997 97 504 505 10.1046/j.1365-2141.1997.d01-3343.x 9163625

[B86] Sazci A Ergul E Kaya G Kara I Genotype and allele frequencies of the polymorphic methylenetetrahydrofolate reductase gene in Turkey Cell Biochem Funct 2005 23 51 54 10.1002/cbf.1132 15386535

[B87] Sehirali S Inal MM Yildirim Y Balim Z Kosova B Karamizrak T Sanci M Topcuoglu N Tinar S Prothrombin G20210A mutation in cases with recurrent miscarriage: a study of the mediterranean population Arch Gynecol Obstet 2005 273 170 173 10.1007/s00404-005-0061-7 16189694

[B88] Antoniadi T Hatzis T Kroupis C Economou-Petersen E Petersen MB Prevalence of factor V Leiden, prothrombin G20210A, and MTHFR C677T mutations in a Greek population of blood donors Am J Hematol 1999 61 265 267 10.1002/(SICI)1096-8652(199908)61:4<265::AID-AJH8>3.0.CO;2-# 10440914

[B89] Pallaud C Stranieri C Sass C Siest G Pignatti F Visvikis S Candidate gene polymorphisms in cardiovascular disease: a comparative study of frequencies between a French and an Italian population Clin Chem Lab Med 2001 39 146 154 10.1515/CCLM.2001.025 11341749

[B90] Margaglione M D'Andrea G Giuliani N Brancaccio V De Lucia D Grandone E De Stefano V Tonali PA Di Minno G Inherited prothrombotic conditions and premature ischemic stroke: sex difference in the association with factor V Leiden Arterioscler Thromb Vasc Biol 1999 19 1751 1756 10397694 10.1161/01.atv.19.7.1751

[B91] Rosendaal FR Doggen CJ Zivelin A Arruda VR Aiach M Siscovick DS Hillarp A Watzke HH Bernardi F Cumming AM Preston FE Reitsma PH Geographic distribution of the 20210 G to A prothrombin variant Thromb Haemost 1998 79 706 708 9569177

[B92] Schwender S Großmann R Keller F High prevalence of factor V Leiden mutation is detected in a north to south axis through Germany Journal of Laboratory Medicine 1997 21 347 352

[B93] Reuner KH Ruf A Grau A Rickmann H Stolz E Jüttler E Druschky KF Patscheke H Prothrombin gene G20210-- > A transition is a risk factor for cerebral venous thrombosis Stroke 1998 29 1765 1769 9731592 10.1161/01.str.29.9.1765

[B94] Junker R Koch HG Auberger K Munchow N Ehrenforth S Nowak-Gottl U Prothrombin G20210A gene mutation and further prothrombotic risk factors in childhood thrombophilia Arterioscler Thromb Vasc Biol 1999 19 2568 2572 10521389 10.1161/01.atv.19.10.2568

[B95] Reuner KH Ruf A Kaps M Druschky KF Patscheke H The mutation C677-- > T in the methylene tetrahydrofolate reductase gene and stroke Thromb Haemost 1998 79 450 451 9493611

[B96] O'Shaughnessy KM Fu B Ferraro F Lewis I Downing S Morris NH Factor V Leiden and thermolabile methylenetetrahydrofolate reductase gene variants in an East Anglian preeclampsia cohort Hypertension 1999 33 1338 1341 10373212 10.1161/01.hyp.33.6.1338

[B97] Croft SA Daly ME Steeds RP Channer KS Samani NJ Hampton KK The prothrombin 20210A allele and its association with myocardial infarction Thromb Haemost 1999 81 861 864 10404757

[B98] Morrison ER Miedzybrodzka ZH Campbell DM Haites NE Wilson BJ Watson MS Greaves M Vickers MA Prothrombotic genotypes are not associated with pre-eclampsia and gestational hypertension: results from a large population-based study and systematic review Thromb Haemost 2002 87 779 785 12038776

[B99] Schneider JA Rees DC Liu YT Clegg JB Worldwide distribution of a common methylenetetrahydrofolate reductase mutation Am J Hum Genet 1998 62 1258 1260 10.1086/301836 9545406 PMC1377093

[B100] Alhenc-Gelas M Arnaud E Nicaud V Aubry ML Fiessinger JN Aiach M Emmerich J Venous thromboembolic disease and the prothrombin, methylene tetrahydrofolate reductase and factor V genes Thromb Haemost 1999 81 506 510 10235429

[B101] Harrington DJ Malefora A Schmeleva V Kapustin S Papayan L Blinov M Harrington P Mitchell M Savidge GF Genetic variations observed in arterial and venous thromboembolism -- relevance for therapy, risk prevention and prognosis Clin Chem Lab Med 2003 41 496 500 10.1515/CCLM.2003.075 12747593

[B102] Sawuła W Banecka-Majkutewicz Z Kadziński L Jakóbkiewicz-Banecka J Wegrzyn G Nyka W Banecki B Homocysteine level and metabolism in ischemic stroke in the population of Northern Poland Clin Biochem 2009 42 442 447 10.1016/j.clinbiochem.2008.12.019 19166826

[B103] Lopaciuk S Bykowska K Kwiecinski H Mickielewicz A Czlonkowska A Mendel T Kuczynska-Zardzewialy A Szelagowska D Windyga J Schröder W Herrmann FH Jedrzejowska H Factor V Leiden, prothrombin gene G20210A variant, and methylenetetrahydrofolate reductase C677T genotype in young adults with ischemic stroke Clin Appl Thromb Hemost 2001 7 346 350 10.1177/107602960100700418 11697722

[B104] Nizankowska-Mogilnicka E Adamek L Grzanka P Domagala TB Sanak M Krzanowski M Szczeklik A Genetic polymorphisms associated with acute pulmonary embolism and deep venous thrombosis Eur Respir J 2003 21 25 30 10.1183/09031936.03.00034302 12570104

[B105] Szperl M Dzielinska Z Roszczynko M Malek LA Makowiecka-Ciesla M Demkow M Kadziela J Prejbisz A Florczak E Zielinski T Januszewicz A Ruzyllo W Genetic variants in hypertensive patients with coronary artery disease and coexisting atheromatous renal artery stenosis Med Sci Monit 2008 14 CR611 616 19043368

[B106] Akcali C Ozkur M Erbagci Z Benlier N Aynacioglu AS Association of insertion/deletion polymorphism of the angiotensin-converting enzyme gene with angio-oedema accompanying chronic urticaria but not chronic urticaria without angio-oedema or the autologous serum skin test response J Eur Acad Dermatol Venereol 2008 22 83 86 18181977 10.1111/j.1468-3083.2007.02353.x

[B107] Comas D Schmid H Braeuer S Flaiz C Busquets A Calafell F Bertranpetit J Scheil HG Huckenbeck W Efremovska L Schmidt H Alu insertion polymorphisms in the Balkans and the origins of the Aromuns Ann Hum Genet 2004 68 120 127 10.1046/j.1529-8817.2003.00080.x 15008791

[B108] Lichter-Konecki U Schlotter M Yaylak C Ozgüç M Coskun T Ozalp I Wendel U Batzler U Trefz FK Konecki D DNA haplotype analysis at the phenylalanine hydroxylase locus in the Turkish population Hum Genet 1989 81 373 376 10.1007/BF00283695 2564839

[B109] Stuhrmann M Riess O Monch E Kurdoglu G Haplotype analysis of the phenylalanine hydroxylase gene in Turkish phenylketonuria families Clin Genet 1989 36 117 121 10.1111/j.1399-0004.1989.tb03173.x 2569949

[B110] Chen SH Giblett ER Anderson JE Fossum BL Genetics of glutamic-pyruvic transaminase: its inheritance, common and rare variants, population distribution, and differences in catalytic activity Ann Hum Genet 1972 35 401 409 5073687

[B111] Moran CN Vassilopoulos C Tsiokanos A Jamurtas AZ Bailey ME Montgomery HE Wilson RH Pitsiladis YP The associations of ACE polymorphisms with physical, physiological and skill parameters in adolescents Eur J Hum Genet 2006 14 332 339 10.1038/sj.ejhg.5201550 16391565

[B112] Biondi G Calabro V Colonna-Romano S Giangregorio M Malaspina P Petrucci R Santolamazza C Santolamazza P Tramontano E Battistuzzi G Common and rare genetic variants of human red blood cell enzymes in Italy Anthropol Anz 1989 47 155 174 2528324

[B113] Dianzani I Devoto M Camaschella C Saglio G Ferrero GB Cerone R Romano C Romeo G Giovannini M Riva E Angeneydt F Trefz FK Okano Y Woo SLC Haplotype distribution and molecular defects at the phenylalanine hydroxylase locus in Italy Hum Genet 1990 86 69 72 10.1007/BF00205176 1979309

[B114] Santovito A Selvaggi A Cervella P Castellano S Bigatti MP Sella G Delpero M Polymorphic Alu insertions in five North-West Italian populations Am J Hum Biol 2007 19 589 592 10.1002/ajhb.20605 17546608

[B115] Aulehla-Scholz C Vorgerd M Sautter E Leupold D Mahlmann R Ullrich K Olek K Horst J Phenylketonuria: distribution of DNA diagnostic patterns in German families Hum genet 1988 78 353 355 10.1007/BF00291734 2896157

[B116] Herrmann FH Wulff K Wehnert M Seidlitz G Guttler F Haplotype analysis of classical and mild phenotype of phenylketonuria in the German Democratic Republic Clin Genet 1988 34 176 180 10.1111/j.1399-0004.1988.tb02859.x 2902943

[B117] Kompf J Ritter H Polymorphism of alanine aminotransferase (E.C.2.7.6.1): common and rare alleles Hum Genet 1979 51 287 292 511157 10.1007/BF00283396

[B118] Schmidt S Schone N Ritz E Association of ACE gene polymorphism and diabetic nephropathy? The Diabetic Nephropathy Study Group Kidney Int 1995 47 1176 1181 10.1038/ki.1995.167 7783416

[B119] Schunkert H Hense HW Holmer SR Stender M Perz S Keil U Lorell BH Riegger GA Association between a deletion polymorphism of the angiotensin-converting-enzyme gene and left ventricular hypertrophy N Engl J Med 1994 330 1634 1638 10.1056/NEJM199406093302302 8177269

[B120] Welch SG Mills PR Gaensslen RE Phenotypic distributions of red cell glutamate-pyruvate transaminase (E.C.2.6.1.2) isoenzymes in British and New York populations Humangenetik 1975 27 59 62 1140814 10.1007/BF00283506

[B121] Narain Y Yip A Murphy T Brayne C Easton D Evans JG Xuereb J Cairns N Esiri MM Furlong RA Rubinsztein DC The ACE gene and Alzheimer's disease susceptibility J Med Genet 2000 37 695 697 10.1136/jmg.37.9.695 10978362 PMC1734696

[B122] Sullivan SE Moore SD Connor JM King M Cockburn F Steinmann B Gitzelmann R Daiger SP Woo SL Haplotype distribution of the human phenylalanine hydroxylase locus in Scotland and Switzerland Am J Hum Genet 1989 44 652 659 2565077 PMC1715636

[B123] de Bosschere JP Dinh Khoi T Quennehen F Le Treut A Fauchet R Le Gall JY Etude du polymorphisme génétique de quelques enzymes érythrocytaires dans la région de Rennes Nouv Rev Fr Hematol 1979 21 9 15 493110

[B124] Abadie V Lyonnet S Melle D Berthelon M Caillaud C Labrune Ph Rey F Rey J Munnich A Molecular basis of phenylketonuria in France Developmental Brain Dysfunction 1993 6 120 126

[B125] Kidd JR Pakstis AJ Zhao H Lu RB Okonofua FE Odunsi A Grigorenko E Bonné-Tamir B Friedlaender J Schulz LO Parnas J Kidd KK Haplotypes and linkage disequilibrium at the phenylalanine hydroxylase locus, PAH, in a global representation of populations Am J Hum Genet 2000 66 1882 1899 10.1086/302952 10788337 PMC1378054

[B126] Solovenchuk LL Polymorphic biochemical systems in the population of immigrant inhabitants of the northeastern USSR. I. The genetic structure and its heterogeneity due to sexual dimorphism and to the duration of separate groups living under extreme environmental conditions Genetika 1983 19 1327 1334 (in Russian) 6414886

[B127] Altukhov Iu P Khil'chevskaia RI Shurkhal AV Polymorphism and heterozygosity levels of the Russian population of Moscow: data for 22 gene loci coding blood proteins Genetika 1981 17 548 555 (in Russian) 7195852

[B128] Nazarov IB Woods DR Montgomery HE Shneider OV Kazakov VI Tomilin NV Rogozkin VA The angiotensin converting enzyme I/D polymorphism in Russian athletes Eur J Hum Genet 2001 9 797 801 10.1038/sj.ejhg.5200711 11781693

[B129] Jaruzelska J Henriksen KF Guttler F Riess O Borski K Blin N Slomski R The codon 408 mutation associated with haplotype 2 is predominant in Polish families with phenylketonuria Hum Genet 1991 86 247 250 10.1007/BF00202402 1671768

[B130] Zygulska M Eigel A Aulehla-Scholz C Pietrzyk JJ Horst J Molecular analysis of PKU haplotypes in the population of southern Poland Hum Genet 1991 86 292 294 10.1007/BF00202412 1671770

[B131] Schlesinger D Dobosz T GPT polymorphism in the Polish population Arch Immunol Ther Exp 1976 24 423 428 962516

[B132] Grzeszczak W Misztalski T Is insertion/deletion polymorphism angiotensin-converting enzyme gene responsible for long-life? Przegl Lek 2002 59 79 83 (in Polish) 12152254

[B133] Shen P Lavi T Kivisild T Chou V Sengun D Gefel D Shpirer I Woolf E Hillel J Feldman MW Oefner PJ Reconstruction of patrilineages and matrilineages of Samaritans and other Israeli populations from Y-chromosome and mitochondrial DNA sequence variation Hum Mutat 2004 24 248 260 10.1002/humu.20077 15300852

[B134] Capelli C Redhead N Romano Calì F Lefranc G Delague V Megarbane A Felice AE Pascali VL Neophytou PI Poulli Z Novelletto A Malaspina P Terrenato L Berebbi A Fellous M Thomas MG Goldstein DB Population structure in the Mediterranean basin: a Y chromosome perspective Ann Hum Genet 2006 70 207 225 10.1111/j.1529-8817.2005.00224.x 16626331

[B135] Zalloua PA Platt DE El Sibai M Khalife J Makhoul N Haber M Xue Y Izaabel H Bosch E Adams SM Arroyo E López-Parra AM Aler M Picornell A Ramon M Jobling MA Comas D Bertranpetit J Wells RS Tyler-Smith C Genographic Consortium Identifying genetic traces of historical expansions: Phoenician footprints in the Mediterranean Am J Hum Genet 2008 83 633 642 10.1016/j.ajhg.2008.10.012 18976729 PMC2668035

[B136] Capelli C Brisighelli F Scarnicci F Arredi B Caglia' A Vetrugno G Tofanelli S Onofri V Tagliabracci A Paoli G Pascali VL Y chromosome genetic variation in the Italian peninsula is clinal and supports an admixture model for the Mesolithic-Neolithic encounter Mol Phylogenet Evol 2007 44 228 239 10.1016/j.ympev.2006.11.030 17275346

[B137] Onofri V Alessandrini F Turchi C Fraternale B Buscemi L Pesaresi M Tagliabracci A Y-chromosome genetic structure in sub-Apennine populations of Central Italy by SNP and STR analysis Int J Legal Med 2007 121 234 237 10.1007/s00414-007-0153-y 17287987

[B138] Francalacci P Morelli L Underhill PA Lillie AS Passarino G Useli A Madeddu R Paoli G Tofanelli S Calò CM Ghiani ME Varesi L Memmi M Vona G Lin AA Oefner P Cavalli-Sforza LL Peopling of three Mediterranean islands (Corsica, Sardinia, and Sicily) inferred by Y-chromosome biallelic variability Am J Phys Anthropol 2003 121 270 279 10.1002/ajpa.10265 12772214

[B139] Semino O Passarino G Oefner PJ Lin AA Arbuzova S Beckman LE De Benedictis G Francalacci P Kouvatsi A Limborska S Marcikiae M Mika A Mika B Primorac D Santachiara-Benerecetti AS Cavalli-Sforza LL Underhill PA The genetic legacy of Paleolithic Homo sapiens sapiens in extant Europeans: a Y chromosome perspective Science 2000 290 1155 1159 10.1126/science.290.5494.1155 11073453

[B140] Wells RS Yuldasheva N Ruzibakiev R Underhill PA Evseeva I Blue-Smith J Jin L Su B Pitchappan R Shanmugalakshmi S Balakrishnan K Read M Pearson NM Zerjal T Webster MT Zholoshvili I Jamarjashvili E Gambarov S Nikbin B Dostiev A Aknazarov O Zalloua P Tsoy I Kitaev M Mirrakhimov M Chariev A Bodmer WF The Eurasian heartland: a continental perspective on Y-chromosome diversity Proc Nat Acad Sci USA 2001 98 10244 10249 10.1073/pnas.171305098 11526236 PMC56946

[B141] Kayser M Lao O Anslinger K Augustin C Bargel G Edelmann J Elias S Heinrich M Henke J Henke L Hohoff C Illing A Jonkisz A Kuzniar P Lebioda A Lessig R Lewicki S Maciejewska A Monies DM Pawłowski R Poetsch M Schmid D Schmidt U Schneider PM Stradmann-Bellinghausen B Szibor R Wegener R Wozniak M Zoledziewska M Roewer L Dobosz T Ploski R Significant genetic differentiation between Poland and Germany follows present-day political borders, as revealed by Y-chromosome analysis Hum Genet 2005 117 428 443 10.1007/s00439-005-1333-9 15959808

[B142] Balanovsky O Rootsi S Pshenichnov A Kivisild T Churnosov M Evseeva I Pocheshkhova E Boldyreva M Yankovsky N Balanovska E Villems R Two sources of the Russian patrilineal heritage in their Eurasian context Am J Hum Genet 2008 82 236 250 10.1016/j.ajhg.2007.09.019 18179905 PMC2253976

[B143] Marjanovic D Fornarino S Montagna S Primorac D Hadziselimovic R Vidovic S Pojskic N Battaglia V Achilli A Drobnic K Andjelinovic S Torroni A Santachiara-Benerecetti AS Semino O The peopling of modern Bosnia-Herzegovina: Y-chromosome haplogroups in the three main ethnic groups Ann Hum Genet 2005 69 757 763 10.1111/j.1529-8817.2005.00190.x 16266413

[B144] Bosch E Calafell F González-Neira A Flaiz C Mateu E Scheil HG Huckenbeck W Efremovska L Mikerezi I Xirotiris N Grasa C Schmidt H Comas D Paternal and maternal lineages in the Balkans show a homogeneous landscape over linguistic barriers, except for the isolated Aromuns Ann Hum Genet 2006 70 459 487 10.1111/j.1469-1809.2005.00251.x 16759179

[B145] Cinnioğlu C King R Kivisild T Kalfoğlu E Atasoy S Cavalleri GL Lillie AS Roseman CC Lin AA Prince K Oefner PJ Shen P Semino O Cavalli-Sforza LL Underhill PA Excavating Y-chromosome haplotype strata in Anatolia Hum Genet 2004 114 127 148 10.1007/s00439-003-1031-4 14586639

[B146] Regueiro M Cadenas AM Gayden T Underhill PA Herrera RJ Iran: tricontinental nexus for Y-chromosome driven migration Hum Hered 2006 61 132 143 10.1159/000093774 16770078

[B147] Abu-Amero KK Hellani A González AM Larruga AM Cabrera VM Underhill PA Saudi Arabian Y-Chromosome diversity and its relationship with nearby regions BMC Genet 2009 10 59 10.1186/1471-2156-10-59 19772609 PMC2759955

[B148] Zalloua PA Xue Y Khalife J Makhoul N Debiane L Platt DE Royyuru AK Herrera RJ Hernanz DF Blue-Smith J Wells RS Comas D Bertranpetit J Tyler-Smith C Genographic Consortium Y-chromosomal diversity in Lebanon is structured by recent historical events Am J Hum Genet 2008 82 873 882 10.1016/j.ajhg.2008.01.020 18374297 PMC2427286

[B149] Flores C Maca-Meyer N Larruga JM Cabrera VM Karadsheh N Gonzalez AM Isolates in a corridor of migrations: a high-resolution analysis of Y-chromosome variation in Jordan J Hum Genet 2005 50 435 441 10.1007/s10038-005-0274-4 16142507

[B150] Cadenas AM Zhivotovsky LA Cavalli-Sforza LL Underhill PA Herrera RJ Y-chromosome diversity characterizes the Gulf of Oman Eur J Hum Genet 2008 16 374 386 10.1038/sj.ejhg.5201934 17928816

[B151] Luis JR Rowold DJ Regueiro M Caeiro B Cinnioğlu C Roseman C Underhill PA Cavalli-Sforza LL Herrera RJ The Levant versus the Horn of Africa: evidence for bidirectional corridors of human migrations Am J Hum Genet 2004 74 532 544 10.1086/382286 14973781 PMC1182266

[B152] Cruciani F Santolamazza P Shen P Macaulay V Moral P Olckers A Modiano D Holmes S Destro-Bisol G Coia V Wallace DC Oefner PJ Torroni A Cavalli-Sforza LL Scozzari R Underhill PA A back migration from Asia to sub-Saharan Africa is supported by high-resolution analysis of human Y-chromosome haplotypes Am J Hum Genet 2002 70 1197 1214 10.1086/340257 11910562 PMC447595

[B153] Arredi B Poloni ES Paracchini S Zerjal T Fathallah DM Makrelouf M Pascali VL Novelletto A Tyler-Smith C A predominantly neolithic origin for Y-chromosomal DNA variation in North Africa Am J Hum Genet 2004 75 338 345 10.1086/423147 15202071 PMC1216069

[B154] Semino O Santachiara-Benerecetti AS Falaschi F Cavalli-Sforza LL Underhill PA Ethiopians and Khoisan share the deepest clades of the human Y-chromosome phylogeny Am J Hum Genet 2002 70 265 268 10.1086/338306 11719903 PMC384897

[B155] Lovell A Moreau C Yotova V Xiao F Bourgeois S Gehl D Bertranpetit J Schurr E Labuda D Ethiopia: between Sub-Saharan Africa and western Eurasia Ann Hum Genet 2005 69 275 287 10.1046/J.1469-1809.2005.00152.x 15845032

[B156] Zietkiewicz E Yotova V Gehl D Wambach T Arrieta I Batzer M Cole DE Hechtman P Kaplan F Modiano D Moisan JP Michalski R Labuda D Haplotypes in the dystrophin DNA segment point to a mosaic origin of modern human diversity Am J Hum Genet 2003 73 994 1015 10.1086/378777 14513410 PMC1180505

[B157] Bourgeois S Yotova V Wang S Bourtoumieu S Moreau C Michalski R Moisan JP Hill K Hurtado AM Ruiz-Linares A Labuda D X-chromosome lineages and the settlement of the Americas Am J Phys Anthropol 2009 140 417 428 10.1002/ajpa.21084 19425105

[B158] Behar DM Metspalu E Kivisild T Achilli A Hadid Y Tzur S Pereira L Amorim A Quintana-Murci L Majamaa K Herrnstadt C Howell N Balanovsky O Kutuev I Pshenichnov A Gurwitz D Bonne-Tamir B Torroni A Villems R Skorecki K The matrilineal ancestry of Ashkenazi Jewry: portrait of a recent founder event Am J Hum Genet 2006 78 487 497 10.1086/500307 16404693 PMC1380291

[B159] Behar DM Metspalu E Kivisild T Rosset S Tzur S Hadid Y Yudkovsky G Rosengarten D Pereira L Amorim A Kutuev I Gurwitz D Bonne-Tamir B Villems R Skorecki K Counting the founders: the matrilineal genetic ancestry of the Jewish Diaspora PLoS One 2008 3 e2062 10.1371/journal.pone.0002062 18446216 PMC2323359

[B160] Irwin J Saunier J Strouss K Paintner C Diegoli T Sturk K Kovatsi L Brandstätter A Cariolou MA Parson W Parsons TJ Mitochondrial control region sequences from northern Greece and Greek Cypriots Int J Legal Med 2008 122 87 89 10.1007/s00414-007-0173-7 17492459

[B161] Babalini C Martínez-Labarga C Tolk HV Kivisild T Giampaolo R Tarsi T Contini I Barać L Janićijević B Martinović Klarić I Pericić M Sujoldzić A Villems R Biondi G Rudan P Rickards O The population history of the Croatian linguistic minority of Molise (southern Italy): a maternal view Eur J Hum Genet 2005 13 902 912 10.1038/sj.ejhg.5201439 15886710

[B162] Ottoni C Martínez-Labarga C vitelli L Scano G Fabrini E Contini I Biondi G Rickards O Human mitochondrial DNA variation in Southern Italy Ann Hum Biol 2009 36 785 811 10.3109/03014460903198509 19852679

[B163] Poetscha M Wittigb H Krause D Lignitz E Mitochondrial diversity of a northeast German population sample Forensic Sci Int 2003 137 125 132 10.1016/j.forsciint.2003.06.001 14609647

[B164] Tetzlaff S Brandstätter A Wegener R Parson W Weirich V Mitochondrial DNA population data of HVS-I and HVS-II sequences from a northeast German sample Forensic Sci Int 2007 172 218 224 10.1016/j.forsciint.2006.12.016 17331686

[B165] Helgason A Hickey E Goodacre S Bosnes V Stefánsson K Ward R Sykes B mtDNA and the Islands of the North Atlantic: Estimating the Proportions of Norse and Gaelic Ancestry Am J Hum Genet 2001 68 723 737 10.1086/318785 11179019 PMC1274484

[B166] Malyarchuk BA Grzybowski T Derenko MV Czarny J Woźniak M Miścicka-Śliwka D Mitochondrial DNA variability in Poles and Russians Ann Hum Genet 2002 66 261 283 10.1046/j.1469-1809.2002.00116.x 12418968

