# Supplementary material for: The origin of Eastern European Jews revealed by autosomal, sex chromosomal and mtDNA polymorphisms
Source: Biol Direct. 2010 Oct 6;5:57. doi: 10.1186/1745-6150-5-57 (PMC2964539; doi:10.1186/1745-6150-5-57)
Supplement: Additional file 1 — Allele frequencies tables, Tables S1-S7. The file contains seven tables that give the allele frequencies of the employed polymorphisms. [file 1745-6150-5-57-S1.DOC]

**Supplementary Table 1 / Allele frequencies of the autosomal protein electrophoretic markers**

|  | N* | AK-1 | AK-2 | N* | ADA-1 | ADA-2 | N* | PGM1-1 | PGM1-2 | N* | PGD-A | PGD-C | N* | ACP-A | ACP-B | ACP-C | N* | ESD-1 | ESD-2 | N* | HP-1 | HP-2 | N* | GC-1F | GC-1S | GC-2 | References |
| --- | --- | --- | --- | --- | --- | --- | --- | --- | --- | --- | --- | --- | --- | --- | --- | --- | --- | --- | --- | --- | --- | --- | --- | --- | --- | --- | --- |
| EEJ | 540 | .941 | .059 | 811 | .909 | .091 | 539 | .737 | .263 | 539 | .979 | .020 | 818 | .279 | .682 | .039 | 180 | .894 | .106 | 1144 | .318 | .682 | 533 | .122 | .595 | .279 | 1 |
| Iraqi Jews | 620 | .960 | .040 | 583 | .846 | .154 | 481 | .681 | .318 | 292 | .983 | .015 | 374 | .311 | .650 | .039 | 317 | .849 | .151 | 602 | .275 | .725 | 110 | .191 | .623 | .182 | 1 |
| Iranian Jews | 182 | .962 | .038 | 182 | .868 | .132 | 183 | .732 | .268 | 178 | .972 | .028 | 228 | .344 | .632 | .024 | 92 | .864 | .136 | 431 | .306 | .694 | 79 | .120 | .608 | .272 | 1 |
| Moroccan Jews | 306 | .967 | .033 | 306 | .897 | .103 | 306 | .618 | .379 | 306 | .938 | .062 | 306 | .232 | .730 | .038 | 110 | .859 | .141 | 304 | .280 | .720 | 117 | .158 | .491 | .350 | 1 |
| Yemenite Jews | 551 | .960 | .040 | 303 | .878 | .122 | 551 | .724 | .276 | 312 | .938 | .063 | 347 | .152 | .789 | .059 | 156 | .788 | .212 | 398 | .312 | .688 | 60 | .167 | .708 | .125 | 1 |
| Ethiopian Jews | 110 | .995 | .005 | 111 | .964 | .036 | 111 | .797 | .194 | 111 | .910 | .090 | 111 | .041 | .959 | 0 | 111 | .829 | .171 | 109 | .385 | .615 | 35 | .429 | .357 | .214 | 1 |
| Palestinians | 707 | .977 | .023 | 557 | .868 | .132 | 709 | .704 | .296 | 506 | .937 | .063 | 522 | .244 | .722 | .034 | 728 | .769 | .231 | 792 | .314 | .686 | 328 | .218 | .598 | .184 | 1 |
| Turks | 545 | .941 | .059 | 536 | .896 | .104 | 349 | .693 | .307 | 519 | .955 | .045 | 504 | .319 | .639 | .042 | 489 | .846 | .154 | 1023 | .298 | .702 | 766 | .173 | .571 | .256 | 58-60 |
| Greeks | 943 | .958 | .042 | 312 | .915 | .085 | 737 | .678 | .322 | 985 | .959 | .039 | 907 | .295 | .650 | .055 | 111 | .869 | .131 | 3755 | .353 | .647 | 301 | .140 | .648 | .213 | 61-63 |
| Italians^1^ | 752 | .965 | .035 | 777 | .919 | .081 | 1663 | .715 | .285 | 1267 | .974 | .026 | 1149 | .286 | .658 | .057 | 1211 | .862 | .138 | 1069 | .361 | .639 | 756 | .157 | .575 | .266 | 1 |
| Germans^2^ | 2083 | .962 | .038 | 1757 | .939 | .061 | 2481 | .772 | .228 | 1100 | .977 | .022 | 2862 | .349 | .595 | .056 | 1024 | .880 | .119 | 4958 | .391 | .609 | 618 | .148 | .572 | .279 | 1 |
| British | 549 | .977 | .023 | 469 | .933 | .067 | 1033 | .769 | .230 | 549 | .979 | .021 | 549 | .332 | .617 | .051 | 128 | .910 | .090 | 586 | .404 | .596 | 1016 | .148 | .568 | .282 | 1 |
| French | 723 | .963 | .037 | 710 | .958 | .042 | 457 | .736 | .264 | 693 | .976 | .024 | 8247 | .320 | .633 | .046 | 525 | .889 | .111 | 1750 | .391 | .609 | 140 | .175 | .571 | .254 | 1(Paris) |
| Russians | 2021 | .964 | .036 | 1047 | .929 | .071 | 5288 | .713 | .287 | 3493 | .961 | .039 | 4776 | .331 | .617 | .052 | 965 | .917 | .083 | 7604 | .362 | .638 | 2071 | .125 | .578 | .297 | 1 |
| Poles^3^ | 1374 | .957 | .043 | 1064 | .940 | .060 | 1449 | .739 | .261 | 858 | .974 | .026 | 2867 | .353 | .565 | .081 | 1826 | .908 | .092 | 9400 | .366 | .634 | 209 | .103 | .581 | .315 | 1 |

* – Number of individuals.

1 – Simple average for 12 Italian localities and provinces.

2 – Simple average for 9 German speaking localities and provinces.

3 –Simple average for 3 Polish provinces.

**Supplementary Table 2 / Allele frequencies of the autosomal markers near the CF locus**

|  | N* | KM19-1 | KM19-2 | N^1^ | J311-1 | J311-2 | N^1^ | metH-1 | metH-2 | N^1^ | metD-1 | metD-2 | References |
| --- | --- | --- | --- | --- | --- | --- | --- | --- | --- | --- | --- | --- | --- |
| EEJ | 139 | .784 | .216 | 125 | .424 | .576 | 130 | .492 | .508 | 123 | .837 | .163 | Unpublished results |
| Iraqi Jews | 47 | .766 | .234 | 46 | .543 | .457 | 49 | .531 | .469 | 47 | .830 | .170 | Unpublished results |
| Iranian Jews | 30 | .763 | .237 | 27 | .444 | .556 | 28 | .643 | .357 | 30 | .833 | .167 | Unpublished results |
| Moroccan Jews | 28 | .821 | .179 | 30 | .467 | .533 | 34 | .618 | .382 | 28 | .857 | .143 | Unpublished results |
| Yemenite Jews | 67 | .701 | .299 | 61 | .361 | .639 | 67 | .532 | .468 | 60 | .817 | .183 | Unpublished results |
| Ethiopian Jews | 54 | .648 | .352 | 42 | .476 | .524 | 54 | .667 | .333 | 54 | .852 | .148 | Unpublished results |
| Palestinians | 44 | .727 | .273 | 37 | .405 | .595 | 42 | .405 | .595 | 44 | .909 | .091 | Unpublished results |
| Turks | 29 | .621 | .379 | 26 | .269 | .731 | 1 | .475 | .525 | 16 | .875 | .125 | 64 |
| Greeks | 43 | .558 | .442 | 84 | .298 | .702 | 80 | .475 | .525 | 84 | .881 | .119 | 65 |
| Italians | 300 | .720 | .280 | 167 | .425 | .575 | 173 | .555 | .445 | 145 | .814 | .186 | 65 |
| Germans | 321 | .698 | .302 | 512 | .424 | .576 | 502 | .566 | .434 | 461 | .809 | .191 | 65-68 |
| British | 507 | .753 | .247 | 372 | .425 | .575 | 127 | .661 | .339 | 104 | .779 | .221 | 69-73 |
| French | 673 | .721 | .279 | 188 | .463 | .537 | 182 | .516 | .484 | 174 | .874 | .126 | 74-77 |
| Russians | 66 | .818 | .182 | 108 | .296 | .704 | 36 | .528 | .472 |  |  |  | 78,79 |
| Poles | 42 | .571 | .429 | 26 | .385 | .615 | 32 | .625 | .375 | 40 | .725 | .275 | 80 |

* – Number of chromosomes.

1 – Greeks.

**Supplementary Table 3 / Allele frequencies of the autosomal thrombophilic polymorphisms**

|  | N* | FV-G | FV-A | N* | FII-G | FII-A | N* | MT-C | MT-T | N* | CBS-D | CBS-I | references |
| --- | --- | --- | --- | --- | --- | --- | --- | --- | --- | --- | --- | --- | --- |
| EEJ | 411 | .982 | .018 | 361 | .974 | .026 | 406 | .539 | .461 | 279 | .953 | .047 | 3,17 |
| Iraqi Jews | 374 | .960 | .040 | 304 | .977 | .023 | 137 | .759 | .241 | 58 | .905 | .095 | 3,17 |
| Iranian Jews | 221 | .991 | .009 | 274 | .993 | .007 | 145 | .686 | .314 | 64 | .906 | .094 | 3,17 |
| Moroccan Jews | 411 | .973 | .027 | 151 | .987 | .013 | 134 | .664 | .336 | 118 | .898 | .102 | 3,17 |
| Yemenite Jews | 425 | .996 | .004 | 355 | .996 | .004 | 161 | .817 | .183 | 65 | .938 | .062 | 3,17 |
| Ethiopian Jews | 54 | .991 | .009 | 229 | 1.00 | 0 | 54 | .852 | .148 | 52 | .875 | .125 | 3,17 |
| Palestinians | 581 | .918 | .082 | 107 | .967 | .033 | 284 | .678 | .322 | 90 | .906 | .094 | 3,17 |
| Turks | 387 | .959 | .041 | 311 | .987 | .013 | 1885 | .675 | .325 | 106 | .925 | .075 | 81-87 |
| Greeks | 160 | .975 | .025 | 160 | .978 | .022 | 160 | .647 | .353 | 25 | .960 | .040 | 17,88 |
| Italians | 1198 | .981 | .019 | 1212 | .979 | .021 | 1198 | .561 | .439 | 519 | .927 | .073 | 17,89-91 |
| Germans | 6279 | .967 | .033 | 724 | .992 | .008 | 182 | .670 | .330 | 1056 | .918 | .082 | 17,92-95 |
| British | 363 | .974 | .026 | 662 | .989 | .011 | 458 | .694 | .306 | 1572 | .919 | .081 | 17,96-99 |
| French | 1877 | .973 | .027 | 398 | .986 | .014 | 1878 | .639 | .361 | 1935 | .918 | .082 | 17,89,100 |
| Russians | 93 | .984 | .016 | 93 | .989 | .011 | 229 | .749 | .251 | 1 | .932 | .068 | 101 |
| Poles | 338 | .981 | .019 | 338 | .991 | .009 | 492 | .687 | .313 | 59 | .932 | .068 | 102-105 |

* – Number of individuals.

1 – Poles.

**Supplementary Table 4 / Allele frequencies of the rest of the autosomal polymorphisms**

|  | N* | PAH-1 | PAH-2 | N** | GPT-1 | GPT-2 | N** | ACE-I | ACE-D | references |
| --- | --- | --- | --- | --- | --- | --- | --- | --- | --- | --- |
| EEJ | 564 | .546 | .454 | 522 | .560 | .440 | 281 | .383 | .617 | 14, Unpublished results |
| Iraqi Jews | 75 | .427 | .573 | 323 | .588 | .412 | 69 | .370 | .630 | 14, Unpublished results |
| Iranian Jews | 67 | .448 | .552 | 73 | .760 | .240 | 64 | .313 | .688 | 14, Unpublished results |
| Moroccan Jews | 65 | .677 | .323 | 173 | .552 | .448 | 59 | .314 | .686 | 14, Unpublished results |
| Yemenite Jews | 72 | .556 | .444 | 190 | .726 | .274 | 53 | .283 | .717 | 14, Unpublished results |
| Ethiopian Jews | 64 | .734 | .266 | 111 | .773 | .227 | 37 | .284 | .716 | 14, Unpublished results |
| Palestinians | 126 | .571 | .429 | 208 | .587 | .413 | 55 | .382 | .618 | 1, Unpublished results |
| Turks | 84 | .464 | .536 | 500 | .551 | .449 | 204 | .417 | .583 | 58,106-109 |
| Greeks | 1 | .511 | .489 | 156 | .558 | .442 | 1045 | .410 | .590 | 62,107,110,111 |
| Italians | 59 | .559 | .441 | 7743 | .535 | .465 | 489 | .378 | .622 | 89,112-114 |
| Germans | 270 | .589 | .411 | 10280 | .523 | .477 | 424 | .440 | .560 | 108,115-119, E kunert personal communication |
| British | 82 | .683 | .317 | 1444 | .528 | .472 | 342 | .472 | .528 | 120-122, LA Tyfield personal communication |
| French | 108 | .685 | .315 | 413 | .544 | .456 | 1480 | .450 | .550 | 89,123,124 |
| Russians | 96 | .646 | .354 | 3891 | .513 | .487 | 449 | .496 | .504 | 125-128 |
| Poles | 130 | .638 | .362 | 703 | .519 | .481 | 689 | .485 | .515 | 105,129-132 |

* – Number of chromosomes.

** – Number of individuals.

1 – Average of Turks and Bulgarians (L Kalaydjieva personal communication).

**Supplementary Table 5 / Haplogroup frequencies of the non-recombining Y chromosome (NRY)**

|  | N | A | B | C | E(xE1b1b1) | E1b1b1 | F(xG,I,J,K) | G | I | J(xJ2) | J2 | K(xP) | P(xR1) | R1(xR1a1) | R1a1 | Ref. |
| --- | --- | --- | --- | --- | --- | --- | --- | --- | --- | --- | --- | --- | --- | --- | --- | --- |
| EEJ | 311 |  |  |  | .006 | .229 | .006 | .100 | .042 | .174 | .206 | .032 | .051 | .090 | .064 | 48,133 |
| Iraqi Jews | 99 |  |  |  |  | .162 |  | .101 |  | .162 | .121 | .131 | .263 | .051 | .010 | 133,54 |
| Iranian Jews | 49 |  |  |  |  | .122 | .082 |  |  | .163 | .122 | .102 | .347 | .020 | .041 | 54 |
| Moroccan Jews | 103 |  |  |  |  | .181 |  | .214 |  | .184 | .146 | .087 | .029 | .194 |  | 133,54 |
| Libyan Jews | 20 |  |  |  |  | .300 |  | .100 |  | .100 | .400 |  |  | .050 | .050 | 133 |
| Jerban Jews | 19 |  |  |  |  | .053 |  | .105^1^ |  | .316 | .526 |  |  |  |  | 134 |
| Yemenite Jews | 94 | .021 |  |  |  | .213 |  | .074 |  | .330 | .149 | .021 | .106 | .064 | .021 | 133,54 |
| Ethiopian Jews | 27 | .481 |  |  | .185 | .259 |  |  |  |  | .037 | .037 |  |  |  | 54 |
| Palestinians | 290 |  |  |  | .010 | .245 |  | .086 | .045 | .345 | .193 | .041 | .003 | .021 | .010 | 135 |
| Italians | 853 |  |  |  | .001 | .138 | .004 | .104 | .063 | .046 | .182 | .040 | .001 | .387 | .026 | 136-138 |
| French | 23 |  |  |  |  | .087 |  |  | .174 | .043 | .130 |  | .043 | .522 |  | 139 |
| British | 25 |  |  |  |  | .040 |  | .080^2^ | .160 |  |  |  |  | .720 |  | 140 |
| Germans | 1215 |  |  | .003^3^ | .002 | .062 |  | .043^2^ | .236 |  | .040 | .032 | .013 | .389 | .179 | 141 |
| Poles | 913 |  |  |  | .005 | .045 |  | .020^2^ | .173 |  | .025 | .043 | .003 | .116 | .570 | 141 |
| Russians | 1228 |  |  | .002 |  | .022 | .005 | .007 | .176 | .005 | .021 | .226 | .006 | .059 | .471 | 142 |
| Serbo-Croats | 256 |  |  |  |  | .145 | .031 | .020 | .531 | .008 | .063 | .027 |  | .039 | .131 | 143 |
| Albanians | 30 |  |  |  |  | .233 | .067 | .033 | .167 | .033 | .167 | .033 |  | .133 | .133 | 144 |
| Greeks | 117 |  |  | .009 | .009 | .214 |  | .034 | .120 | .009 | .205 | .026 |  | .222 | .154 | 139,144 |
| Macedonians | 52 |  |  |  |  | .231 |  | .038 | .288 |  | .115 | .038 |  | .154 | .135 | 144 |
| Romanians | 67 |  |  |  | .015 | .134 | .015 | .104 | .403 | .015 | .119 |  |  | .119 | .075 | 144 |
| Turks | 523 | .004 |  | .013 | .006 | .107 | .006 | .109 | .054 | .092 | .243 | .107 | .029 | .163 | .069 | 145 |
| Iranians-North | 33 |  |  | .030 |  |  |  | .152 |  | .091 | .242 | .121 | .121 | .212 | .030 | 146 |
| Iranians-South | 117 |  | .026 |  | .017 | .052 | .043 | .128 |  | .120 | .231 | .103 | .034 | .085 | .162 | 146 |
| Iraqis | 203 |  |  |  | .010 | .108 | .005 | .025 | .005 | .310 | .266 | .089 |  | .113 | .069 | 147 |
| Cypriots | 165 |  |  |  | .012 | .310 | .006 | .133 | .006 | .152 | .230 | .006 | .024 | .115 | .006 | 135 |
| Syrians | 208 |  |  |  | .034 | .130 |  | .058 | .043 | .226 | .293 | .087 | .010 | .048 | .072 | 135 |
| Lebanese | 914 |  |  | .001 | .014 | .152 |  | .066 | .048 | .201 | .259 | .056 | .087 | .081 | .025 | 148 |
| Jordanians | 101 | .010 |  |  |  | .178 | .010 | .059 | .050 | .406 | .158 | .010 | .020 | .079 | .020 | 149 |
| Saudi-Arabs | 157 |  | .019 | .013 | .089 | .076 | .019 | .032 |  | .420 | .159 | .076 | .025 | .019 | .051 | 147 |
| Qataris | 72 |  | .028 |  | .083 | .056 | .014 | .028 |  | .583 | .083 |  | .042 | .014 | .069 | 150 |
| United Arab Emirates | 164 |  |  | .012 | .055 | .116 | .079 | .043 |  | .348 | .104 | .049 | .061 | .049 | .073 | 150 |
| Omanis | 121 | .008 |  | .033 | .091 | .141 | .017 | .017 |  | .380 | .099 | .099 | .008 | .017 | .091 | 151 |
| Yemenites | 62 |  |  |  | .032 | .129 |  | .016 |  | .726 | .097 |  |  |  |  | 150 |
| Egyptians | 147 | .027 |  |  | .027 | .354 |  | .088 | .007 | .197 | .122 | .082 | .020 | .048 | .027 | 151 |
| Moroccans | 113 | .018 |  |  | .035 | .779 |  | .035 |  | .044 | .080 |  |  | .009 |  | 152 |
| Algeriams | 54 |  |  |  | .019 | .555 |  | .111^1^ |  | .204 | .037 | .019 |  | .056 |  | 153 |
| Tunisians | 148 |  |  |  | .020 | .493 |  | .047^1^ |  | .324 | .034 | .007 | .007 | .068 |  | 153 |
| Ethiopians-Oromo | 78 | .103 | .013 |  | .167 | .628 |  |  |  | .026 | .013 | .051 |  |  |  | 154 |
| Ethiopians-Amhara | 48 | .146 | .021 |  | .104 | .354 |  |  |  | .313 | .021 | .042 |  |  |  | 154 |

1 – All F*(xH,I,J,K) are assumed to belong to G.

2 – All F*(xI,J2,K) are assumed to belong to G.

3 – Four unassingned chromosomes are assumed to belong to C.

**Supplementary Table 6 / Haplogroup frequencies of the dystrophin locus, dys44, on Xp21.3***

|  |  | B | B | B | B | B | B | B | B | B | B | B | B | B | B | B | B | B | B | B | b | b | b | b | B | B | B | b | b | B | B | B | b | B | b | b | b | B | B | B | B | B | B | B | B | B | B | B | B | B | b | B | B | B | B | b | B | B | B | B | B | B |
| --- | --- | --- | --- | --- | --- | --- | --- | --- | --- | --- | --- | --- | --- | --- | --- | --- | --- | --- | --- | --- | --- | --- | --- | --- | --- | --- | --- | --- | --- | --- | --- | --- | --- | --- | --- | --- | --- | --- | --- | --- | --- | --- | --- | --- | --- | --- | --- | --- | --- | --- | --- | --- | --- | --- | --- | --- | --- | --- | --- | --- | --- | --- |
|  |  | 0 | 0 | 0 | 0 | 0 | 0 | 0 | 0 | 0 | 0 | 0 | 0 | 0 | 0 | 0 | 0 | 0 | 0 | 0 | 0 | 0 | 0 | 0 | 0 | 0 | 0 | 0 | 0 | 0 | 1 | 1 | 1 | 1 | 1 | 1 | 1 | 1 | 1 | 1 | 1 | 1 | 0 | 0 | 0 | 0 | 0 | 0 | 1 | 1 | 1 | 1 | 1 | 0 | 0 | 0 | 1 | 1 | 1 | 0 | 0 | 1 |
|  |  | 0 | 0 | 0 | 0 | 0 | 0 | 0 | 1 | 1 | 1 | 2 | 2 | 3 | 3 | 3 | 3 | 3 | 3 | 5 | 5 | 6 | 7 | 8 | 8 | 5 | 8 | 8 | 8 | 9 | 2 | 2 | 2 | 2 | 3 | 3 | 3 | 3 | 3 | 3 | 3 | 3 | 0 | 1 | 3 | 3 | 6 | 6 | 0 | 0 | 3 | 6 | 6 | 1 | 7 | 8 | 0 | 6 | 6 | 9 | 4 | 0 |
|  | **N^1^** | 1 | 2 | 3 | 4 | 5 | 6 | 8 | 1 | 3 | 6 | 4 | 5 | 0 | 1 | 3 | 4 | 5 | 9 | 1 | 2 | 8 | 1 | 0 | 1 | 8 | 2 | 4 | 9 | 0 | 6 | 7 | 8 | 9 | 0 | 1 | 2 | 4 | 5 | 6 | 7 | 8 | 7 | 0 | 2 | 6 | 3 | 7 | 6 | 7 | 3 | 6 | 7 | 2 | 0 | 6 | 4 | 5 | 9 | 7 | 1 | 8 |
| EEJ | 110 | .400 | .109 | .318 |  | .036 | .055 | .018 | .018 | .018 |  |  |  |  |  |  |  |  |  |  |  |  |  |  |  |  |  | .009 | .009 |  |  |  |  |  |  |  |  |  |  |  | .009 |  |  |  |  |  |  |  |  |  |  |  |  |  |  |  |  |  |  |  |  |  |
| Iraqi J | 27 | .630 | .037 | .074 |  | .037 | .037 | .074 | .037 |  |  |  |  |  |  | .037 |  |  |  | .037 |  |  |  |  |  |  |  |  |  |  |  |  |  |  |  |  |  |  |  |  |  |  |  |  |  |  |  |  |  |  |  |  |  |  |  |  |  |  |  |  |  |  |
| Iranian J | 21 | .429 | .048 | .095 |  | .048 | .048 | .190 |  |  |  | .048 | .048 |  |  |  |  |  |  |  |  |  |  |  |  |  |  |  |  |  |  |  |  |  |  |  |  | .048 |  |  |  |  |  |  |  |  |  |  |  |  |  |  |  |  |  |  |  |  |  |  |  |  |
| Moroccan J | 29 | .552 | .034 | .172 |  |  | .069 | .034 | .034 |  | .034 |  |  |  |  | .034 |  |  |  |  |  |  |  |  |  |  |  |  |  |  |  |  |  |  |  |  |  |  |  | .034 |  |  |  |  |  |  |  |  |  |  |  |  |  |  |  |  |  |  |  |  |  |  |
| Yemenite J | 31 | .452 | .161 | .065 |  |  |  | .129 | .065 |  | .032 |  |  |  |  |  |  | .032 |  |  |  |  |  |  |  |  |  |  |  |  |  |  |  |  |  |  |  |  | .032 |  |  | .032 |  |  |  |  |  |  |  |  |  |  |  |  |  |  |  |  |  |  |  |  |
| Ethiopian J | 16 | .375 | .188 | .063 |  | .063 | .063 |  |  |  |  |  |  |  |  |  |  |  |  |  |  |  |  |  |  |  |  |  |  |  | .063 | .063 | .063 | .063 |  |  |  |  |  |  |  |  |  |  |  |  |  |  |  |  |  |  |  |  |  |  |  |  |  |  |  |  |
| Bulgarian J | 18 | .500 |  | .111 |  | .056 | .222 | .056 | .056 |  |  |  |  |  |  |  |  |  |  |  |  |  |  |  |  |  |  |  |  |  |  |  |  |  |  |  |  |  |  |  |  |  |  |  |  |  |  |  |  |  |  |  |  |  |  |  |  |  |  |  |  |  |
| Turkish J^2^ | 12 | .333 | .167 | .250 |  | .083 |  | .083 |  |  | .083 |  |  |  |  |  |  |  |  |  |  |  |  |  |  |  |  |  |  |  |  |  |  |  |  |  |  |  |  |  |  |  |  |  |  |  |  |  |  |  |  |  |  |  |  |  |  |  |  |  |  |  |
| Palestinians | 23 | .391 | .043 | .087 |  |  | .217 | .087 |  |  |  |  |  |  |  |  |  |  |  |  |  |  |  |  |  | .043 |  |  |  |  |  |  |  |  | .043 | .043 | .043 |  |  |  |  |  |  |  |  |  |  |  |  |  |  |  |  |  |  |  |  |  |  |  |  |  |
| Poles | 30 | .333 | .067 | .267 |  |  | .100 | .067 | .033 | .033 |  |  |  |  |  |  | .033 |  | .033 |  | .033 |  |  |  |  |  |  |  |  |  |  |  |  |  |  |  |  |  |  |  |  |  |  |  |  |  |  |  |  |  |  |  |  |  |  |  |  |  |  |  |  |  |
| Italians | 54 | .426 | .037 | .296 |  |  | .074 | .056 | .037 |  | .019 |  |  |  | .019 |  |  |  |  |  |  | .019 |  |  |  |  |  |  |  |  |  |  |  |  |  |  |  |  |  |  |  |  |  |  |  |  |  |  |  |  | .019 |  |  |  |  |  |  |  |  |  |  |  |
| Germans | 73 | .342 | .041 | .260 |  | .027 | .137 | .027 | .027 |  |  |  | .027 | .014 |  |  | .027 |  |  |  |  |  | .014 | .014 | .014 |  |  |  |  | .027 |  |  |  |  |  |  |  |  |  |  |  |  |  |  |  |  |  |  |  |  |  |  |  |  |  |  |  |  |  |  |  |  |
| Basques | 41 | .268 | .049 | .244 | .024 | .073 | .171 | .049 |  | .024 |  |  |  |  | .024 |  |  |  |  |  |  |  |  |  |  |  | .024 |  |  |  |  |  |  |  |  |  |  |  |  |  |  |  |  |  |  |  |  |  |  |  |  | .024 | .024 |  |  |  |  |  |  |  |  |  |
| Spaniards | 55 | .382 | .073 | .182 |  | .073 | .109 | .036 | .018 |  |  |  |  |  |  |  |  |  |  |  |  |  |  |  |  |  |  |  |  |  |  |  |  |  |  |  |  |  |  | .018 |  |  |  |  |  |  |  |  |  |  |  |  |  | .018 | .018 | .018 | .018 | .018 | .018 |  |  |  |
| French | 129 | .341 | .016 | .209 |  | .109 | .140 | .070 | .031 | .008 | .023 |  |  |  |  | .023 |  |  |  | .008 |  |  |  |  |  |  |  |  |  |  |  |  |  |  |  |  |  |  |  |  |  |  | .008 |  |  |  |  |  |  |  |  |  |  |  | .016 |  |  |  |  |  |  |  |
| Bretons^3^ | 151 | .298 | .079 | .245 |  | .073 | .132 | .053 | .040 | .020 |  |  |  |  |  |  |  |  |  | .013 |  |  | .013 |  |  |  |  |  |  |  |  |  |  |  |  |  |  |  |  |  |  |  |  |  |  |  |  |  |  |  |  |  |  |  | .007 |  |  |  |  | .013 | .007 |  |
| E^4^ Oromo | 19 | .158 |  | .263 |  | .053 |  | .105 | .053 |  |  |  |  |  |  |  |  |  |  |  |  |  |  |  |  | .053 |  |  |  |  |  |  |  |  |  |  |  |  |  |  |  |  |  | .053 | .105 | .053 |  | .053 |  |  |  |  |  |  |  |  |  |  |  |  |  | .053 |
| E^4^ Amhara | 30 | .267 | .133 | .167 |  |  | .033 | .100 | .033 |  | .033 |  |  |  |  |  |  |  |  |  |  |  |  |  |  |  |  |  |  |  |  |  |  |  |  |  |  |  |  |  |  |  | .067 | .067 |  |  | .033 |  | .033 | .033 |  |  |  |  |  |  |  |  |  |  |  |  |

* – References 155-157.

1 – Number of chromosomes.

2 – D Labuda personal communication.

3 – Haplogroup 32 also registered with a frequency of 0.007.

4 – Ethiopians.

**Supplementary Table 7 / Haplogroup frequencies of mtDNA**

|  | N | L0 | L1 | L2 | L3 | L4 | L5 | L6 | M | M1 | C | D | G | N | N1 | N1a | N1b | N1c | I | A | W | X | Y | R | U | U1 | U2 | U3 | U4 | U5 | U6 | U7 | K | U8 | U9 | R0a | HV | HV1 | HV2 | H | HV0 | J | T | F | B | Ref |
| --- | --- | --- | --- | --- | --- | --- | --- | --- | --- | --- | --- | --- | --- | --- | --- | --- | --- | --- | --- | --- | --- | --- | --- | --- | --- | --- | --- | --- | --- | --- | --- | --- | --- | --- | --- | --- | --- | --- | --- | --- | --- | --- | --- | --- | --- | --- |
| EEJ | 337 |  |  | .018 |  |  |  |  | .009 | .006 |  |  |  | .003 |  |  | .077 |  | .009 |  | .021 | .003 |  |  |  | .012 | .006 | .003 | .006 | .024 | .006 | .015 | .291 |  |  | .033 | .021 | .059 | .003 | .199 | .039 | .095 | .045 |  |  | 158 |
| Iraqi Jews | 135 |  |  |  |  |  |  |  |  | .022 |  |  |  |  |  |  | .022 |  | .044 |  | .104 | .007 |  | .022 |  |  | .007 | .111 |  |  |  | .022 | .022 | .015 |  |  |  |  | .037 | .119 | .007 | .222 | .215 |  |  | 159 |
| Iranian Jews | 82 |  |  |  |  |  |  |  | .012 |  |  |  |  | .037 |  |  |  |  |  |  | .012 | .049 |  |  |  | .061 | .012 | .024 | .012 |  |  | .098 |  |  |  | .012 |  |  |  | .305 |  | .159 | .207 |  |  | 159 |
| Moroccan Jews | 149 |  | .007 | .007 |  |  |  |  |  |  |  |  |  |  |  |  | .007 |  | .060 |  |  | .060 |  | .007 |  | .007 |  | .007 | .020 | .020 | .013 |  | .154 |  |  | .007 | .007 | .067 |  | .409 | .034 | .094 | .013 |  |  | 159 |
| Libyan Jews | 83 |  |  |  | .036 |  |  |  |  |  |  |  |  | .024 |  |  |  |  |  |  |  | .398 |  |  |  | .012 |  |  |  | .024 |  | .012 | .012 |  |  | .048 | .024 |  |  | .325 |  | .048 | .024 |  |  | 159 |
| Tunisian Jews | 37 |  |  |  | .027 |  |  |  |  |  |  |  |  |  |  |  |  |  | .054 |  |  | .162 |  |  |  |  |  |  | .081 | .027 | .027 |  | .027 |  |  | .081 |  | .027 |  | .405 | .054 |  | .027 |  |  | 159 |
| Bulgarian Jews | 71 |  |  |  |  |  |  |  |  |  |  |  |  | .014 |  |  | .056 |  | .028 |  |  | .056 |  | .014 | .014 |  |  |  | .028 | .042 | .028 |  | .155 | .028 |  | .028 | .014 |  |  | .296 |  | .070 | .127 |  |  | 159 |
| Turkish Jews | 123 |  |  |  |  |  |  |  |  |  |  |  |  | .016 |  |  | .016 |  |  |  |  | .079 |  | .008 | .016 | .024 |  | .008 | .024 | .008 | .008 |  | .063 | .008 |  |  | .008 | .016 |  | .441 | .039 | .118 | .103 |  |  | 159 |
| Yemenite Jews | 119 |  |  |  | .101 | .050 |  | .017 | .008 | .008 |  |  |  |  |  |  |  |  | .017 |  | .008 |  |  | .126 |  | .050 |  | .034 |  |  |  |  | .050 |  |  | .151 |  | .143 | .034 | .034 |  | .151 | .016 |  |  | 159 |
| Ethiopian Jews | 29 | .172 |  | .103 | .138 | .034 | .069 | .034 |  | .172 |  |  |  |  |  | .034 |  |  |  |  | .034 |  |  |  |  | .034 | .034 |  |  |  | .034 |  |  |  |  | .103 |  |  |  |  |  |  |  |  |  | 159 |
| Palestinians | 110 |  |  | .045 | .055 |  |  |  | .036 | .009 |  | .009 | .009 |  |  |  | .100 |  | .027 |  |  | .009 |  |  | .009 | .045 |  | .009 | .018 | .055 | .018 |  | .082 | .009 |  | .073 | .027 | .055 |  | .127 | .018 | .055 | .100 |  |  | 159 |
| Turks | 383 |  |  |  |  |  |  |  | .016 | .003 | .008 | .008 |  | .005 |  | .013 | .008 | .003 | .023 | .005 | .039 | .047 |  | .023 |  | .037 | .010 | .055 | .010 | .055 |  | .016 | .063 | .008 |  | .013 | .018 | .018 | .005 | .253 | .003 | .110 | .123 | .003 |  | 54 |
| Greeks | 318 |  |  |  |  |  |  |  | .009 |  | .003 |  |  |  | .003 | .003 | .003 |  | .025 |  | .025 | .038 |  | .006 |  | .019 | .003 | .038 | .044 | .047 | .006 | .006 | .050 |  |  | .022 | .006 | .006 |  | .418 | .028 | .104 | .085 |  |  | 160 |
| Italians | 1137 |  | .003 |  | .007 | .001 |  |  | .001 | .004 | .001 | .001 |  | .002 |  | .001 | .014 | .004 | .015 |  | .024 | .028 |  | .008 | .002 | .009 | .018 | .018 | .020 | .035 | .006 | .009 | .088 | .002 |  | .010 | .039 | .003 |  | .409 | .037 | .068 | .116 |  |  | 54,161,162 |
| Germans | 485 |  |  |  |  |  |  |  | .004 |  | .004 |  | .002 |  |  |  | .002 |  | .031 |  | .023 | .014 | .002 | .002 | .010 | .002 | .004 | .006 | .037 | .068 |  |  | .054 |  |  | .014 |  |  |  | .472 | .033 | .103 | .109 |  | .002 | 163,164 |
| French | 1240 |  |  |  |  |  |  |  |  |  |  |  |  |  |  |  | .003 |  | .023 |  | .021 | .007 |  |  |  | .001 | .016 | .007 | .022 | .091 |  | .001 | .095 |  |  | .004 | .019 | .002 |  | .471 | .050 | .074 | .093 |  |  | 54 |
| British | 429 |  |  |  |  |  |  |  | .005 |  |  |  |  |  |  |  |  |  | .030 | .002 | .016 | .009 |  |  |  |  | .007 | .007 | .016 | .065 |  |  | .061 |  |  |  |  |  |  | .522 | .037 | .145 | .077 |  |  | 165 |
| Russians | 201 |  |  |  |  |  |  |  | .005 |  |  | .005 | .005 |  |  |  |  |  | .025 |  | .020 | .035 |  | .005 |  | .010 | .015 | .010 | .035 | .104 |  | .005 | .030 |  |  | .005 | .020 |  |  | .423 | .055 | .080 | .109 |  |  | 166 |
| Poles | 436 |  |  |  | .002 |  |  |  | .002 |  | .009 | .002 | .005 |  |  |  | .002 | .002 | .018 |  | .037 | .018 |  | .005 | .002 |  | .009 | .005 | .050 | .087 |  | .002 | .034 | .005 |  |  | .009 |  |  | .452 | .048 | .078 | .115 |  |  | 166 |
| Spaniards | 215 |  | .014 | .009 |  |  |  |  |  | .023 |  | .009 |  | .005 |  |  |  |  | .019 | .005 | .028 | .019 |  | .019 | .005 | .005 | .023 | .014 | .009 | .070 | .009 |  | .074 |  |  |  | .009 |  |  | .395 | .070 | .074 | .074 |  | .019 | 54 |
| Cypriots | 183 |  | .038 |  | .005 |  |  |  |  | .022 | .005 |  |  | .005 |  |  | .011 | .011 | .022 |  | .005 | .060 |  |  |  | .022 | .011 | .044 | .033 | .022 | .005 | .011 | .104 | .016 |  | .027 | .027 |  | .005 | .339 |  | .077 | .071 |  |  | 54 |
| Lebanese | 168 |  |  | .012 | .018 |  |  |  |  | .012 | .024 | .012 |  | .006 |  |  | .036 | .012 | .048 | .006 | .018 | .060 |  |  |  | .054 | .030 | .042 | .006 | .048 | .024 |  | .060 | .006 |  | .024 | .024 | .018 |  | .244 | .006 | .048 | .107 |  |  | 54 |
| Syrians | 215 | .005 | .014 | .028 | .033 | .023 |  |  | .019 |  |  |  |  | .009 |  |  | .028 |  | .042 | .005 | .047 | .019 |  | .005 |  | .047 |  | .093 |  | .009 | .005 | .009 | .047 | .009 |  | .028 | .028 | .009 | .009 | .181 | .005 | .130 | .116 |  |  | 54 |
| Iranians | 560 | .002 |  | .002 | .016 |  |  |  | .039 | .002 | .005 | .004 | .004 | .013 |  | .005 | .005 | .007 | .020 |  | .020 | .023 |  | .045 |  | .025 | .014 | .032 | .011 | .029 | .002 | .082 | .068 | .009 |  | .014 | .066 | .007 | .018 | .170 | .005 | .143 | .093 |  | .005 | 54 |
| Jordanians | 198 |  | .025 | .025 | .070 |  |  |  |  | .020 |  |  |  | .030 |  |  | .015 |  | .010 |  | .015 | .020 |  | .010 |  | .056 | .015 | .040 | .025 |  | .010 | .030 | .045 | .005 |  | .045 | .025 | .020 |  | .202 | .020 | .061 | .157 |  |  | 54 |
| Saudi-Arabians | 319 | .013 | .003 | .031 | .044 | .003 |  |  | .025 | .041 |  |  |  |  |  | .019 | .034 | .028 | .013 |  | .016 | .016 |  |  |  | .016 | .013 | .016 | .003 | .006 | .013 | .009 | .044 | .013 | .009 | .144 | .025 | .006 |  | .125 |  | .219 | .053 |  |  | 54 |
| Yemenites | 118 | .068 |  | .068 | .178 | .025 |  | .127 | .076 | .008 |  |  |  |  |  | .068 | .008 |  |  |  |  | .017 |  | .025 |  | .017 | .025 | .008 | .017 |  |  | .008 | .093 |  |  | .034 | .017 |  |  | .042 |  | .059 | .008 |  |  | 54 |
| Egyptians | 188 | .027 | .016 | .069 | .080 |  | .005 | .005 |  | .074 |  |  | .005 | .005 |  | .005 | .037 |  | .032 |  | .016 | .027 |  | .016 |  | .016 |  | .043 | .005 | .011 | .027 | .016 | .037 |  |  | .053 | .021 | .048 |  | .117 | .011 | .069 | .106 |  |  | 54 |
| Moroccan Arabs | 363 |  | .066 | .083 | .132 |  |  |  |  | .041 |  |  |  |  |  |  | .017 |  |  |  | .003 | .025 |  | .011 |  | .011 | .003 | .008 | .008 | .044 | .077 | .003 | .044 | .008 |  | .006 | .017 |  |  | .242 | .047 | .050 | .055 |  |  | 54 |
| Moroccan Berbers | 319 |  | .047 | .078 | .066 | .003 |  |  |  | .028 |  |  |  |  |  |  | .003 |  |  |  |  | .009 |  | .006 |  | .006 | .003 | .013 | .006 | .060 | .066 |  | .050 | .009 |  | .006 | .013 | .003 |  | .285 | .085 | .075 | .078 |  |  | 54 |
| Ethiopians | 270 | .078 | .022 | .144 | .170 | .067 | .030 | .007 |  | .170 |  |  |  |  |  | .022 |  |  | .011 |  | .011 | .007 |  |  |  | .004 | .007 | .007 |  |  | .030 |  | .015 |  |  | .104 | .026 | .019 |  | .007 |  | .019 | .022 |  |  | 54 |
